# Modeling Site-Specific Nucleotide Biases Affecting Himar1 Transposon Insertion Frequencies in TnSeq Data Sets

**DOI:** 10.1128/mSystems.00876-21

**Published:** 2021-10-19

**Authors:** Sanjeevani Choudhery, A. Jacob Brown, Chidiebere Akusobi, Eric J. Rubin, Christopher M. Sassetti, Thomas R. Ioerger

**Affiliations:** a Department of Computer Science and Engineering, Texas A&M University, College Station, Texas, USA; b Department of Immunology and Infectious Diseases, Harvard School of Public Health, Boston, Massachusetts, USA; c Department of Microbiology and Physiological Systems, University of Massachusetts Medical School, Worcester, Massachusetts, USA; University of California, Berkeley

**Keywords:** Himar1 transposon, TnSeq, gene essentiality, linear regression models, machine learning, mutant fitness, nucleotide patterns, statistical analysis

## Abstract

TnSeq is a widely used methodology for determining gene essentiality, conditional fitness, and genetic interactions in bacteria. The Himar1 transposon is restricted to insertions at TA dinucleotides, but otherwise, few site-specific biases have been identified. As a result, most analytical approaches assume that insertions are expected to be randomly distributed among TA sites in nonessential regions. However, through analysis of Himar1 transposon libraries in Mycobacterium tuberculosis, we demonstrate that there are site-specific biases that affect the frequency of insertion of the Himar1 transposon at different TA sites. We use machine learning and statistical models to characterize patterns in the nucleotides surrounding TA sites that correlate with high or low insertion counts. We then develop a quantitative model based on these patterns that can be used to predict the expected counts at each TA site based on nucleotide context, which can explain up to half of the variance in insertion counts. We show that these insertion preferences exist in Himar1 TnSeq data sets from other mycobacterial and nonmycobacterial species. We present an improved method for identification of essential genes, called TTN-Fitness, that can better distinguish true biological fitness effects by comparing observed counts to expected counts based on our site-specific model of insertion preferences. Compared to previous essentiality methods, TTN-Fitness can make finer distinctions among genes whose disruption causes a fitness defect (or advantage), separating them out from the large pool of nonessentials, and is able to classify many smaller genes (with few TA sites) that were previously characterized as uncertain.

**IMPORTANCE** When using the Himar1 transposon to create transposon insertion mutant libraries, it is known that the transposon is restricted to insertions at TA dinucleotide sites throughout the genome, and the absence of insertions is used to infer which genes are essential (or conditionally essential) in a bacterial organism. It is widely assumed that insertions in nonessential regions are otherwise random, and this assumption is used as the basis of several methods for statistical analysis of TnSeq data. In this paper, we show that the nucleotide sequence surrounding TA sites influences the magnitude of insertions, and these Himar1 insertion preferences (sequence biases) can partially explain why some sites have higher counts than others. We use this predictive model to make improved estimates of the fitness effects of genes, which help make finer distinctions of the phenotype and biological consequences of disruption of nonessential genes.

## INTRODUCTION

TnSeq has become a popular tool for evaluating gene essentiality in bacteria under various conditions (1). The most widely used transposons for bacterial TnSeq are those in the *mariner* family, such as Himar1 (2). To date, it has generally been assumed that the Himar1 transposon, frequently used to generate the transposon libraries, inserts randomly at TA dinucleotide sites in nonessential regions across the genome (3). The abundance of transposon insertions at each TA site can be quantified efficiently using next-generation sequencing (4). Genes or loci with an absence of insertions are considered to be essential, as disruption in these regions is not tolerated (2). Genes or loci with a reduced mean insertion count are considered mutants with growth defects, as disruptions in these regions are not fatal but cause growth impairments or fitness defects (5). Genes that have significant changes in mean counts between conditions are deemed conditionally essential (6).

There are several sources of noise in TnSeq experiments, including stochastic variations in the library generation process as well as instrument and sampling error in DNA sequencing, resulting in a high variability in insertion counts. Statistical methods developed thus far to assess gene essentiality typically assume that insertions occur randomly at TA sites in nonessential regions, and the reason some sites have more insertions than others is largely due to stochastic differences in abundance in the library. However, some studies suggest that transposon insertions at nonessential sites are influenced by the surrounding nucleotides or genomic context. Transposons Tn*5* and Mu (not restricted to TA dinucleotides) showed a bias toward insertions in GC-rich regions and resulted in a less uniform distribution of insertions in the A-T-rich genome (61% AT) of Candida glabrata than their notably less-biased counterpart Tn*7* (7). In addition, Lampe et al. (3) showed that local bendability of the DNA strand can affect the probability of Himar1 insertion at different chromosomal locations in Escherichia coli. Furthermore, an analysis of 14 independent transposon libraries in Mycobacterium tuberculosis H37Rv identified a local sequence pattern around certain TA sites that was nonpermissive for Himar1 insertions [(CG)GnTAnC(CG)] (8). This sequence pattern extended to ∼9% of sites in nonessential regions which almost always had counts of zero (8).

In this paper, we use statistical and machine learning models to identify patterns in the nucleotides surrounding TA sites associated with high and low insertion counts. We discover nucleotide biases within a ±4-bp window around the TA site that suppress Himar1 insertions and other patterns that appear to select for them (i.e., associated with high insertion counts). We capture these biases in a predictive model of Himar1 insertion preferences that can be used to predict expected insertion counts at any TA site as a function of the surrounding nucleotide context. We demonstrate that these insertion preferences exist in other Himar1 TnSeq data sets from M. tuberculosis, as well as other mycobacterial and nonmycobacterial species. The final predictive model explains about half of the variance in insertion counts, presuming the rest comes from stochastic variability between libraries or is due to sampling differences during sequencing. We demonstrate that this model can be used to make improvements in quantifying various degrees of fitness caused by disruption in genes (which are not absolutely essential) by comparing the observed counts to expected counts using a site-specific model of insertion preferences.

## RESULTS

### Insertion counts at TA sites are correlated between libraries.

**FIG 1:**
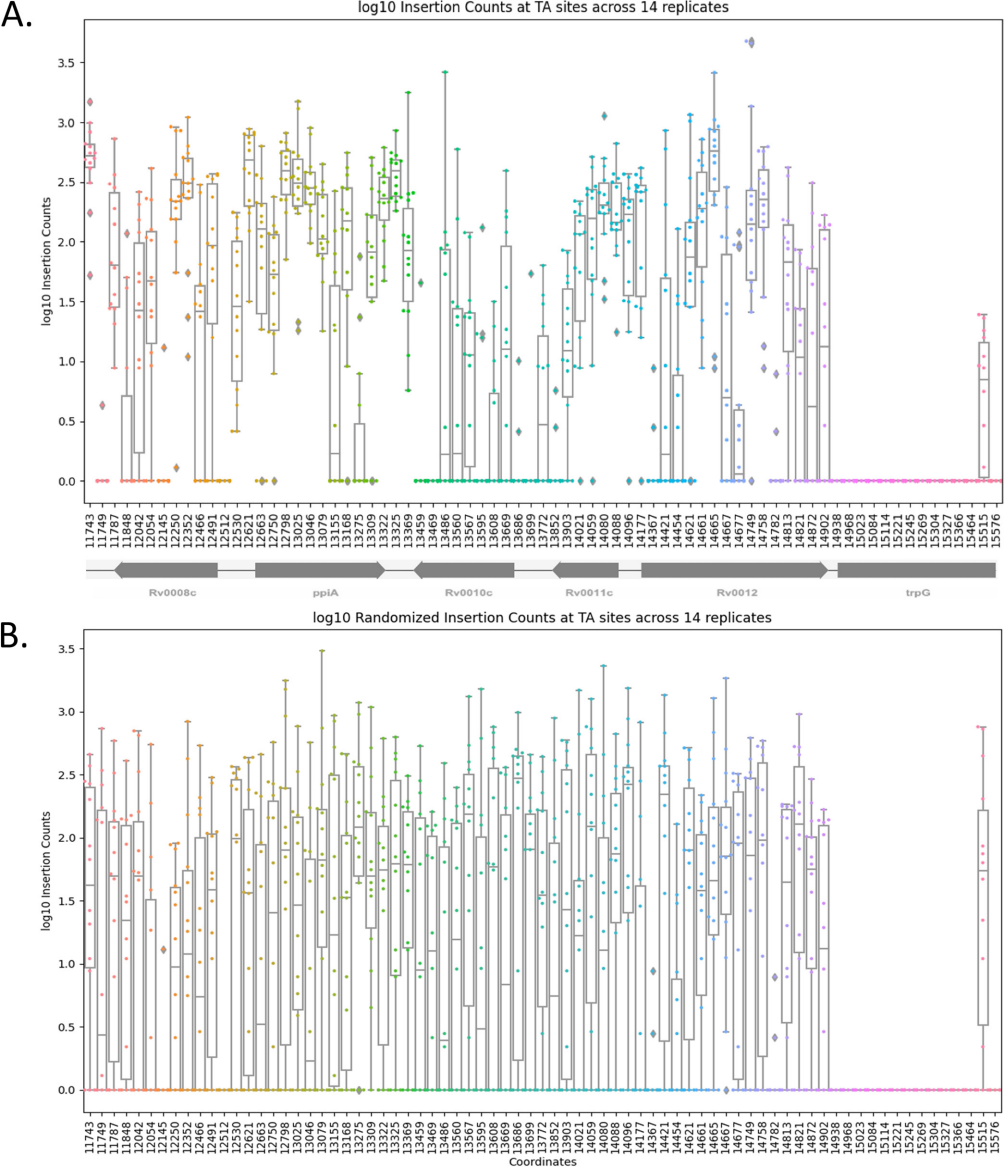
Insertion counts across 14 H37Rv libraries in a region spanning 75 consecutive TA sites. In panel A, a point is plotted for insertion counts at each coordinate for each replicate. This scatterplot is then overlaid with a box-and-whisker plot reflecting the mean and range of insertion counts at each site. The region includes *trpG* for comparison, which is an essential gene, and hence, insertion counts are 0 in this gene. In the nonessential genes, the insertion counts vary more between TA sites than within, supporting that some TA sites have a higher propensity for insertions than others. Panel B shows the same 75 sites after randomizing the insertion counts at all TA sites except those marked Essential and those showing the nonpermissive pattern. The mean and range of counts at each nonessential TA site are much more uniform when randomized.

Variability in insertion counts at TA sites can be attributed to various sources, including abundance in library, experimental randomness, and local sequence biases, as well as genuine biological significance (fitness effects). To attempt to differentiate these, we reanalyzed a previously published collection of 14 independent Himar1 TnSeq libraries grown in standard laboratory medium (8). An extended HMM (hidden Markov model) analysis of the 14 data sets suggests that approximately 11.6% of the organism’s TA sites are essential for growth, and insertions in approximately 3.5% of the sites can cause a growth defect (8). In addition, 9% of sites in nonessential regions have few to no insertions due to a nonpermissive sequence pattern (DeJesus et al. [8]). Insertions at TA sites in regions other than these are generally expected to occur randomly. If true, the insertion counts at the same TA site in different libraries would be expected to be uncorrelated on average. However, our analysis of the 14 H37Rv transposon (Tn) libraries shows that there is substantial correlation of counts at individual TA sites, suggesting that some TA sites have a higher propensity for Himar1 insertion than others. Figure 1 shows the distribution of log_10_ insertion counts from each library in a genomic region with 75 TA sites (the log of counts was taken to better fit a Gaussian distribution). Each library was TTR normalized (trimmed-total read-count normalization) to make counts from data sets of different total size comparable (9). Figure 1A shows that mean insertion counts differ widely among nonessential TA sites, and the variability between TA sites is more than that within each site. Thus, high counts at a TA site in one library tend to have high counts in other libraries, and similarly, sites with low counts occur symmetrically across the libraries. As a comparison, insertion counts at TA sites (excluding those marked essential or following the nonpermissive pattern) were randomized within each library. Figure 1B shows the same 75 consecutive TA sites in this randomized data set. When randomized, the distribution of counts at sites in nonessential regions is much more uniform. The average variance of all insertions within a TA site is 0.430, significantly lower (*P* value < 0.001) than the variance of 0.929 found in the randomized data set. This makes it evident that the correlation of log insertion counts across libraries is greater than expected. In fact, pairwise correlations (measured using Pearson correlation coefficients) of the randomized data sets range from 0.15 to 0.33, averaging to 0.28. Pairwise correlations of 14 libraries are considerably higher, ranging from 0.5 to 0.97, averaging to 0.62. All 90 pairwise correlations had significant *P* values (<0.01) by a permutation test. A significant high correlation across libraries suggests there are site-specific influences, in addition to those previously observed, on insertion probabilities at different TA sites.

### Modeling insertion counts using linear regression.

**FIG 2:**
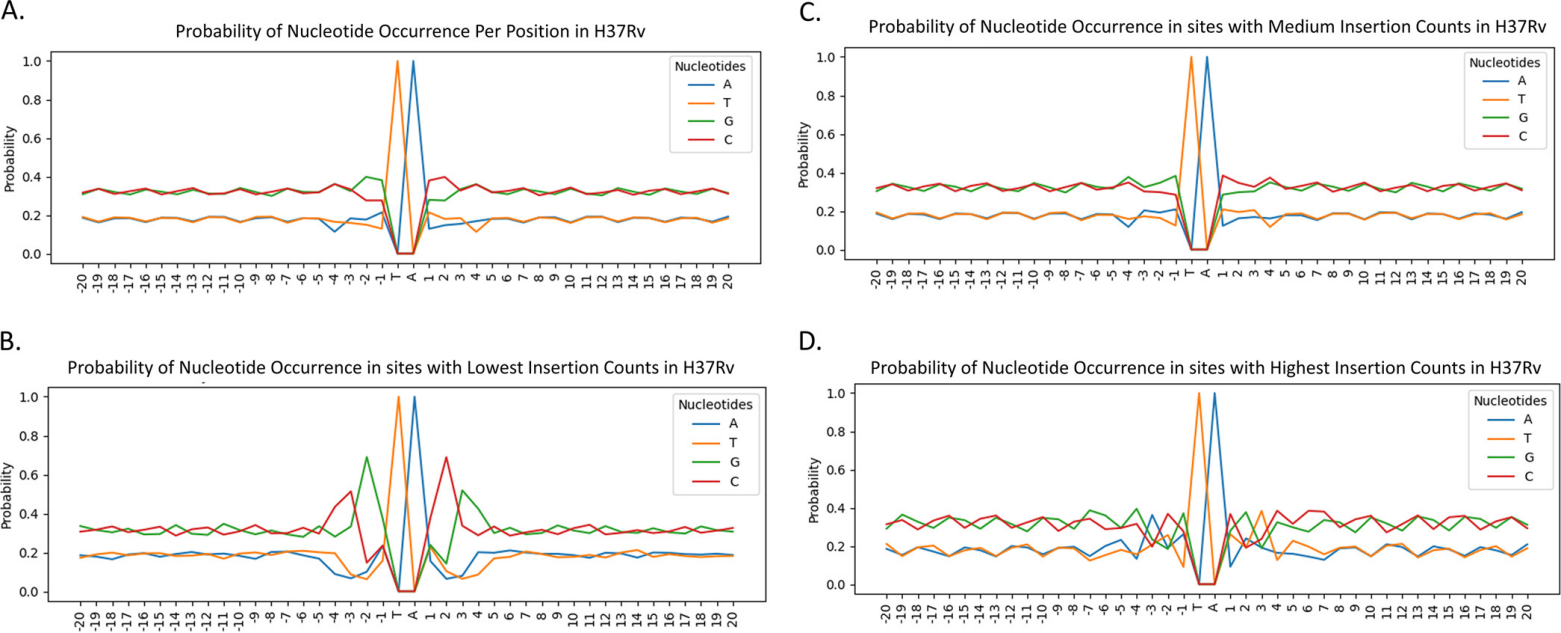
Nucleotide probabilities at positions −20…+20 from the TA site, for three ranges of insertion counts. Panel A shows the nucleotide probability at every position across all ∼65,000 TA sites in nonessential regions. Panel B shows the nucleotide probability across the 6,573 sites in the lowest 10th percentile of the range of insertion counts (lowest 10th percentile insertion counts less than 2.1), Panel C shows the pattern across 52,026 sites in the middle range of insertion counts (greater than 2.1 and less than 291.3), and panel D shows the pattern across 6,505 sites in the highest 10th percentile of the range of insertion counts (90th percentile, insertion counts greater than 291.3).

To determine whether the nucleotides surrounding a TA site influence the probability of insertion, we examined the association of proximal nucleotides on insertion counts, averaged over all nonessential TA sites in the genome. Figure 2 presents evidence of site-specific nucleotide effects that influence the relative abundance of insertions at TA sites. Insertion counts were normalized per library before taking the log. Figure 2A shows overall nucleotide probabilities ±20 bp from the TA site. Most of the deviation in nucleotide probabilities occurs within 4 bp of the central TA site, with probabilities varying up to 20% for some nucleotides. Further insight can be gained by dividing the TA sites into three ranges: sites with lowest counts, sites with medium counts, and sites with highest counts. Figure 2B, depicting the 10th percentile of the range of insertion counts, shows an increase in probabilities of nucleotides C and G, consistent with the nonpermissiveness pattern (8), and a decrease in probabilities of nucleotides ‘A’ and ‘T’ especially at positions ±2 and ±3. Figure 2D, depicting the 90th percentile of insertion counts, also shows drastic changes in nucleotide probability, with a notable increase in propensity for ‘A’ at −3 and ‘T’ at +3. These observations suggest a correlation between the magnitude of insertion counts and nucleotides surrounding TA sites. Thus, insertion counts at a TA site could be affected by the surrounding nucleotides.

**FIG 3:**
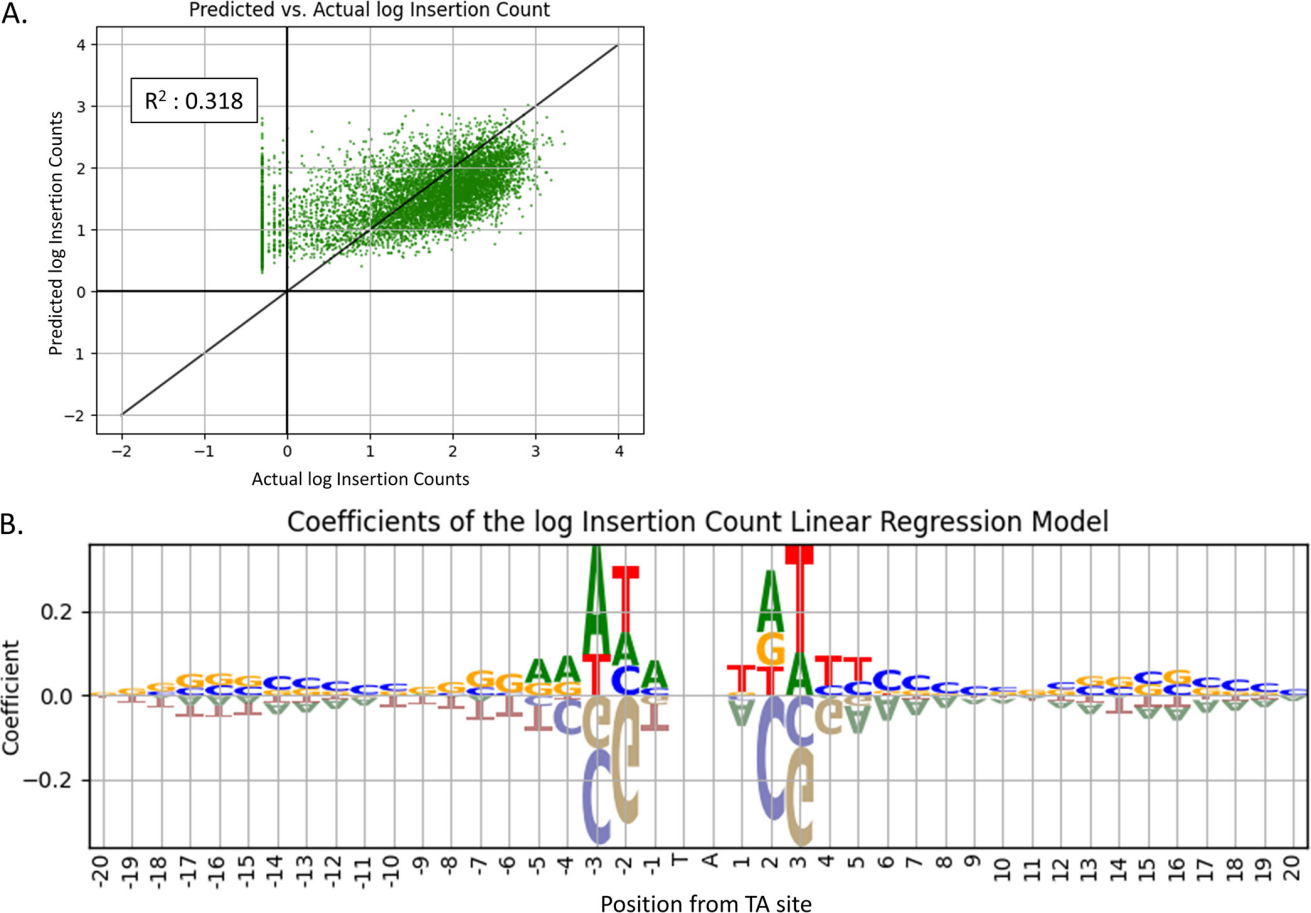
Coefficients and accuracy assessment of linear regression model trained on nucleotides as covariates. Panel A shows predicted counts versus actual log insertion counts using linear regression. The average predictive power of the linear regression model trained with one-hot-encoded nucleotides as the input and log insertion counts as the output using 10-fold cross-validation. The predictive power is moderate (*R*^2^ = 0.318), meaning it is able to explain 31% of the variation in insertion counts based on surrounding nucleotides. Panel B shows coefficients from the trained linear model. The coordinates along the *x* axis give the positions relative to, but not including, the TA site. The model is trained on one-hot-encoded nucleotides and a target value of log insertion counts. The symmetry of the pattern is visible in positions −4, −3, −2, −1 and +1, +2, +3, +4. The nonpermissive pattern (CG)GnTAnC(CG) is visible in this window, as well as high coefficients associated with ‘A’ and ‘T’.

We trained a linear regression model on the 40 nucleotides surrounding the TA site (positions −20…+20) to predict insertion counts in known nonessential regions (67,670 TA sites) using the mean counts from the 14 libraries of H37Rv. The input to the model was a one-hot-encoding of the nucleotides, where each nucleotide at each position was represented by 4 bits and concatenated into a bit vector, totaling 160 binary features. The resulting linear model was

$$\log_{10}\left(\mathrm{InsertionCount}\right)\;=\;w_{0}\;+\;\underset{i\;=\;-20..+20}{\sum }\underset{j\;=\;A,C,G,T}{\sum }w_{\mathrm{ij}}\;\mathrm{nu}c_{\mathrm{ij}}$$

where nuc*_ij_* = 1 if nuc[*i*] = *j*, nuc[*i*] is the nucleotide at position *i* relative to the TA site, and weights *w_ij_* correspond to each of the 160 binary features. This formula is equivalent to a dot-product of a 160-bit vector (plus an intercept) with a vector of weights, log_10_ (insertion count) = *w*_0_ + *w*_1_ *b*_−20=A_ + *w*_2_ *b*_−20=C_ + *w*_3_ *b*_−20=T_ + *w*_4_ *b*_−20=G_ +…+ *w*_157_ *b*_+20=A_ + *w*_158_ *b*_+20=C_ + *w*_159_ *b*_+20=T_ + *w*_160_ *b*_+20=G_, where every four bits encode the nucleotide at a position ±20 bp from the TA site. The model was trained and evaluated using 10-fold cross-validation. Figure 3 shows the average correlation between predicted and observed log_10_ insertion counts. The model has some predictive power (*R*^2^ value of 0.32) but also has high variance. A slight bias can be seen in the figure, where the low counts are predicted too high, and the high counts are predicted too low. This is a consequence of the regression model making predictions that do not span as wide of a range as the actual data, due to inaccurate predictions for the sites with the most extreme values (largest or smallest counts). The accuracy of predictions made by this initial simplified regression will increase with improved models (below), and thus, this effect will be reduced.

In Fig. 3B, nucleotides with highest coefficients in the trained model are located within a window of ±4 bp around the TA site. The pattern created by the nucleotides of these coefficients is consistent with the nonpermissive pattern (CG)GnTAnC(CG) previously reported (8). Nucleotide ‘G’ has the highest absolute coefficient value in the −2 position, and ‘C’ has the highest absolute coefficient value in the +2 position. Moreover, both ‘C’ and ‘G’ have similarly high absolute coefficients in the −3 and +3 positions. In addition to the confirmation of the nonpermissive pattern (large negative coefficients for ‘G’ at −3 and ‘C’ at +3), the figure shows nucleotides ‘A’ and ‘T’ with relatively high positive coefficients in positions −3 and +3 from the TA site. These patterns reinforce the observations made in Fig. 1 and provide further evidence of previously undetected site-specific nucleotide biases that affect Himar1 insertion counts.

### Prediction of insertion counts at TA sites relative to local average counts.

We assume that insertion counts are proportional to the permissiveness of a site, i.e., a site with a less permissive pattern will have lower counts than a site with a more permissive pattern. However, insertion counts are also affected by biological fitness. It is likely that a TA site with a specific nucleotide pattern in a fitness-defect gene will have a lower insertion count than a TA site with the same pattern in a nonessential gene. But this effect (decrease or increase in counts) should be shared by multiple TA sites locally. We can compare the insertion count observed at a site to the observed counts at other TA sites in the region, the level of which should reflect the general fitness effect of disrupting the gene. Thus, modeling this relative (or local) change in insertion counts would allow us to factor out biological effects on counts and focus on the effect of nucleotide patterns on the insertion counts.

This change in insertion counts is quantified for every TA site as a log-fold change (LFC) value. The local average was calculated for each site by taking the mean insertion counts from the previous 5 and next 5 TA sites from the site of interest (i.e., using a sliding window of 11 consecutive TA sites).

$$\mathrm{LocalAverage}\left(i\right)=\frac{1}{10}\left[\overset{i-1}{\underset{i-5}{\sum }}I\mathrm{nsertionCount}\left(i\right)\;+\overset{i+5}{\underset{i+1}{\sum }}I\mathrm{nsertionCount}\left(i\right)\right]$$

The local mean excludes the central site itself and any locations marked as essential during preprocessing. The LFC for each TA site was calculated by taking the log insertion count at that site plus a pseudocount of 10 (to smooth out high variability of LFCs for sites with low counts) and dividing it by the local average:

$$\mathrm{LFC}\left(i\right)={\text{log}}_{2}\left(\frac{\mathrm{InsertionCount}\left(i\right)\;+\;10}{\mathrm{LocalAverage}\left(i\right)\;+\;10}\right)$$

As with the previous model, this linear model was trained and tested using 10-fold cross-validation. The resulting model (see Fig. S2 in the supplemental material) has an average *R*^2^ value of 0.38, indicating that training the model to predict changes in insertion counts (relative to local mean) rather than absolute insertion counts greatly reduces the noise due to local fitness effects (e.g., in a gene where insertion cause growth defects, systematically reducing abundance of insertions in the region). This allows the model to better capture the effect of nucleotides surrounding TA sites on Himar1 insertion preferences.

10.1128/mSystems.00876-21.1 FIG S1 Heatmap of correlation of wig files. The correlation of log insertion counts in the 14-replicate wig files using the Pearson correlation coefficient. Download FIG S1, TIF file, 1.9 MB. Copyright © 2021 Choudhery et al. 2021 Choudhery et al. https://creativecommons.org/licenses/by/4.0/ This content is distributed under the terms of the Creative Commons Attribution 4.0 International license.

10.1128/mSystems.00876-21.2 FIG S2 Coefficients from linear model trained using nucleotides in all 40 positions to predict LFCs. Panel A shows predicted counts versus actual LFC using linear regression. The average predictive power of the linear regression model trained with one-hot-encoded nucleotides in 20 bp from the TA site as the input and LFCs as the output using 10-fold cross-validation. The predictive power was not much higher than the previous insertion counts model, but the variance has decreased, indicating a better model. Panel B shows coefficients from the trained model. The coordinates along the *x* axis give the positions relative to, but not including, the TA site. A symmetric pattern is visible in positions −4, −3, −2, −1 and +1, +2, +3, +4. The nonpermissiveness pattern (CG)GnTAnC(CG) is visible in this window (low values for ‘G’ and ‘C’ at ±2 and 3) as well as high coefficients associated with ‘A’ and ‘T’. Download FIG S2, TIF file, 2.3 MB. Copyright © 2021 Choudhery et al. 2021 Choudhery et al. https://creativecommons.org/licenses/by/4.0/ This content is distributed under the terms of the Creative Commons Attribution 4.0 International license.

### A neural network model explains up to 50% of the variability in insertion counts.

As they can capture nonlinear patterns, neural networks are considered to be some of the most powerful predictors in machine learning (10). To see if we could increase the accuracy of our model, we tried using our data to train a fully connected multilayer feed-forward neural network. The model contained one hidden layer of 50 nodes. This parameter along with other hyperparameters of the network was tuned using a grid search (see details in Materials and Methods) using a random subset of 70% of the data. The remaining 30% of the data was used to test the final hyperparameters. The input to the model consisted of bit-vectors encoding nucleotides surrounding each TA site in the data set, totaling to 160 features. The target value was LFCs (log fold changes of insertion counts relative to local mean). The model performed better than the previous models with an average *R*^2^ of 0.493 (see Fig. S3). Thus, the neural network can explain around half of the variability in insertion counts at TA sites based on surrounding nucleotides; presumably, the remaining differences in counts still reflect stochastic differences in abundance between libraries (or other influences on TA insertion preferences for which we have not yet accounted). However, as is typical for neural networks, this model (as a matrix of connection weights) does not provide us much insight into nucleotide patterns that led to the predictions for the TA sites.

10.1128/mSystems.00876-21.3 FIG S3 Predicted LFC versus actual LFC using feed forward neural network. The input to this linear model was all the one-hot-encoded nucleotides, and the target value as the LFCs. Using 10-fold cross-validation on 70% of the data set, we found the ideal parameters: ‘activation’: ‘tanh’, ‘alpha’: 0.05, ‘early_stopping’: True, ‘hidden_layer_sizes’: (100), ‘learning_rate’: ‘constant’, ‘max_iter’: 500, and ‘solver’: ‘adam’. We tested these hyperparameters on the remaining 30% of the test data and got a fairly high-performing model. We applied these hyperparameters and assessed the model’s fit to the data by performing a 10-fold cross-validation of the entire data set. This yielded an average predictive power (i.e., *R*^2^) that was higher than the previous insertion counts model and LFC model, and the variance has decreased, indicating a better model. Download FIG S3, TIF file, 2.9 MB. Copyright © 2021 Choudhery et al. 2021 Choudhery et al. https://creativecommons.org/licenses/by/4.0/ This content is distributed under the terms of the Creative Commons Attribution 4.0 International license.

### Certain nucleotides surrounding TA sites are associated with high or low insertion frequencies.

**FIG 4:**
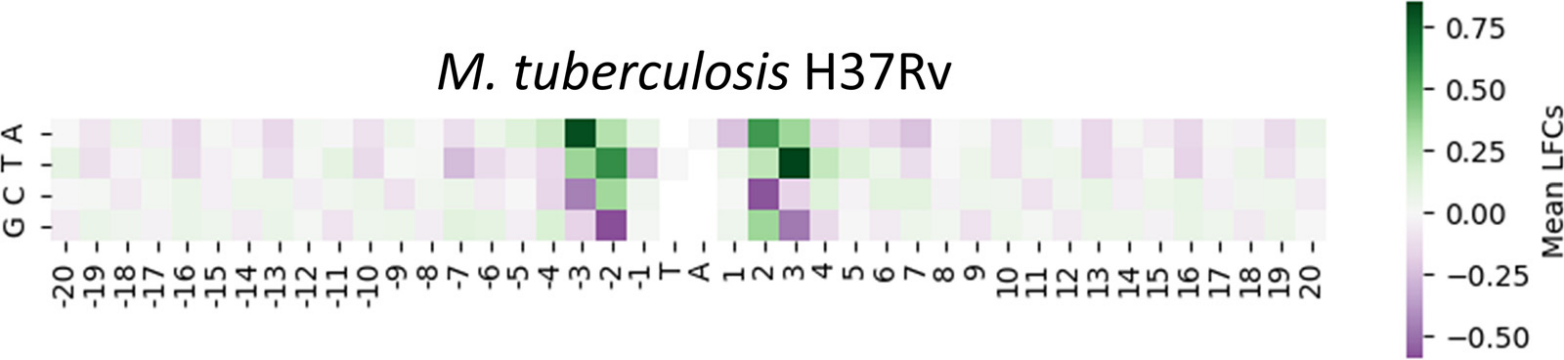
Enrichment and depletion of insertions as a function of nucleotides surrounding TA sites for the 14 libraries of H37Rv *in vitro*. The mean LFC [log fold change: log_2_(observed_count/local_mean)] of sites with each filtered nucleotide at every position in a 20-bp window of the TA site in the H37Rv data set is visualized here. The heatmap is centered at the median of mean LFCs. Nucleotides colored green at certain positions are enriched (have higher than average insertion counts), while those colored purple are depleted (relative to the local average nucleotide content).

It has been previously noted that there are biases in distributions of nucleotides surrounding TA sites, making them more permissive or less permissive. If a site has a pattern that is considered more permissive, it should have a higher insertion count than its neighbors and thus a positive LFC. The opposite is true for sites with a less permissive pattern. They should have lower counts than their neighbors and thus negative LFCs. The heatmap in Fig. 4 was generated to visualize any additional nucleotide biases that may result in unusually high or low insertion counts. For each nucleotide N and position P within ±20 bp of a TA site, the mean LFC was calculated over the subset of TA sites having nucleotide N at position P (Materials and Methods). The heatmap reinforces the idea illustrated in Fig. 2 of the correlation between nucleotide biases and insertion count magnitudes. A ‘G’ in position −2 and its symmetric counterpart ‘C’ in position +2, as well as ‘C’ in the −3 position and its counterpart ‘G’ in the +3 position, are associated with low mean LFCs. This indicates that TA sites with at least one of these nucleotides in their relative positions tend to have lower insertion counts than their neighbors, consistent with the nucleotide bias represented by the nonpermissive pattern (CG)GnTAnC(CG) observed in reference 8. Similarly, there is a distinctive pattern for positive mean LFCs: an ‘A’ in position −3 and its counterpart ‘T’ in position +3 are both associated with higher mean LFCs and hence can be interpreted as being more permissive for Himar1 insertions (associated with increased counts). However, the effects of multiple biases appearing in a single sequence are not additive. For instance, a ‘C’ in the −2 and an ‘A’ in the −3 position do not “cancel” each other out; they are interdependent. We quantify how effects like these combine in the tetranucleotide model below.

There appears to be a slight periodic pattern of the G and C nucleotides surrounding the TA site, between 20 and 4 bp from the TA site in Fig. 4 (also evident in Fig. 2). The nucleotides show an increase in mean LFC for every third position in the sequence. Representing this pattern in a simplistic manner and comparing it to the LFC target variable showed little correlation. Thus, this periodic sequence was not incorporated in our model.

### Symmetric tetranucleotide linear model (STLM).

To gain more insight into the nucleotide patterns observed through the heatmaps, we devised a variant of the linear model, called the symmetric tetranucleotide linear model (STLM). In the linear models previously mentioned, the pattern associated with an individual nucleotide was implicitly assumed to be additive, and thus, each nucleotide position was treated as an independent variable. But we wondered whether a stronger pattern might be found through combinations of these nucleotide positions, which can represent nonlinear interactions.

Training the linear model to predict LFCs based only on the nucleotides in a window of ±4 bp from the TA site yielded nearly identical results to the regression predicting LFCs using all 40 nucleotides (*R*^2^ = 0.35 and the same coefficient pattern for nucleotides in range −4…+4), indicating that most of the influence on LFC predictions is within an 8-bp window (see Fig. S4). This is reinforced by the heatmaps, as a majority of the apparent effects occur within 4 bp from the TA site. If we use all the sequence combinations of the nucleotides in positions −4…+4 as features in our model, we will have 4^8^ = 65,536 features (i.e., terms in a linear model, or inputs to a neural network). However, the patterns of nucleotide biases are symmetrical (reverse-complement), as shown by the heatmaps, thus making the distinction between all 8 nucleotides unnecessary. The 4 nucleotides upstream of the TA appear to affect the insertion counts in the same way as the reverse-complement of the 4 nucleotides downstream of the TA site. Therefore, it is necessary only to capture the association of 4 nucleotides at a time on LFCs in the model. Hence, we shift to training our models based on combinations of 4 nucleotides, i.e., tetranucleotides, which reduces the number of features in our model to 4^4^ = 256.

10.1128/mSystems.00876-21.4 FIG S4 LFC prediction using linear regression with only nucleotides in −4…+4 positions from the TA site. Panel A shows predicted counts versus actual LFC using linear regression. The average predictive power of the linear regression model trained with one-hot-encoded nucleotides in 4 bp from the TA site as the input and LFCs as the output using 10-fold cross-validation. The predictive power is moderate (*R*^2^ = 0.352), meaning it can explain 35% of the variation in insertion counts based on surrounding nucleotides, not much different than the LFC linear model trained using all 40 nucleotides, indicating the nucleotides in this window are very important. Panel B shows coefficients from the trained model. The coordinates along the *x* axis give the positions relative to, but not including, the TA site. These coefficients are almost identical to the relative magnitudes of the nucleotides in the −4…+4 window of the linear model for predicting LFCs, as well as the log insertion count linear model. Download FIG S4, TIF file, 2.5 MB. Copyright © 2021 Choudhery et al. 2021 Choudhery et al. https://creativecommons.org/licenses/by/4.0/ This content is distributed under the terms of the Creative Commons Attribution 4.0 International license.

**FIG 5:**
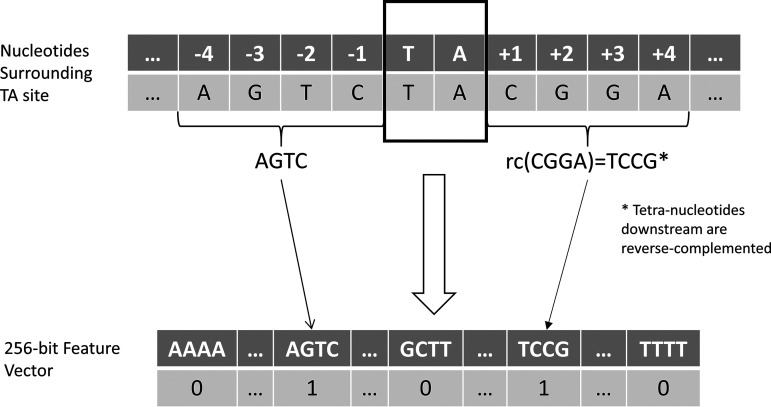
Illustration of the STLM. For each TA site the upstream tetranucleotide and reverse-complemented (rc) downstream tetranucleotide are extracted. The relative bits are set in a 256-bit vector that is given as an input to the STLM to predict LFCs.

As input to the STLM, each TA site is represented as a vector where all features are set to 0 except for the upstream tetranucleotide and reverse-complemented downstream tetranucleotide (Fig. 5). This is essentially the same as adding two bit-vectors, one vector with the bit for the upstream tetranucleotide on and another separate vector with the bit for the downstream tetranucleotide on. The result is a sparse 256-bit vector with only 2 bits on (except when the two tetranucleotides are the same, in which case the single feature value for the tetranucleotide is set to 2). The result is a linear model that follows the equation

$$\mathrm{LFC}=\mathrm{intercept}\;+\;w_{1}b_{\mathrm{AAAA}}\;+\ldots +\;w_{256}b_{\mathrm{TTTT}}$$

where *w*_1_…*w*_256_ are the weights associated with tetranucleotides (to be trained by the model) and *b*_AAAA_…*b*_TTTT_ are the bits corresponding to the presence of the adjacent tetranucleotide features for every TA site. Encoding both the upstream and reverse-complemented downstream tetranucleotides allows us to use the same model to represent the bias from both sides of the TA simultaneously as independent features, additively contributing equal weight. Assume for a given TA site, both upstream and downstream tetranucleotides are associated with high LFCs; then, they will reinforce to predict an even higher insertion count for that site. But if the upstream tetranucleotide has a trend to contribute a high LFC and the reverse-complemented downstream tetranucleotide has a trend to contribute a low LFC, they will tend to cancel each other out.

**FIG 6:**
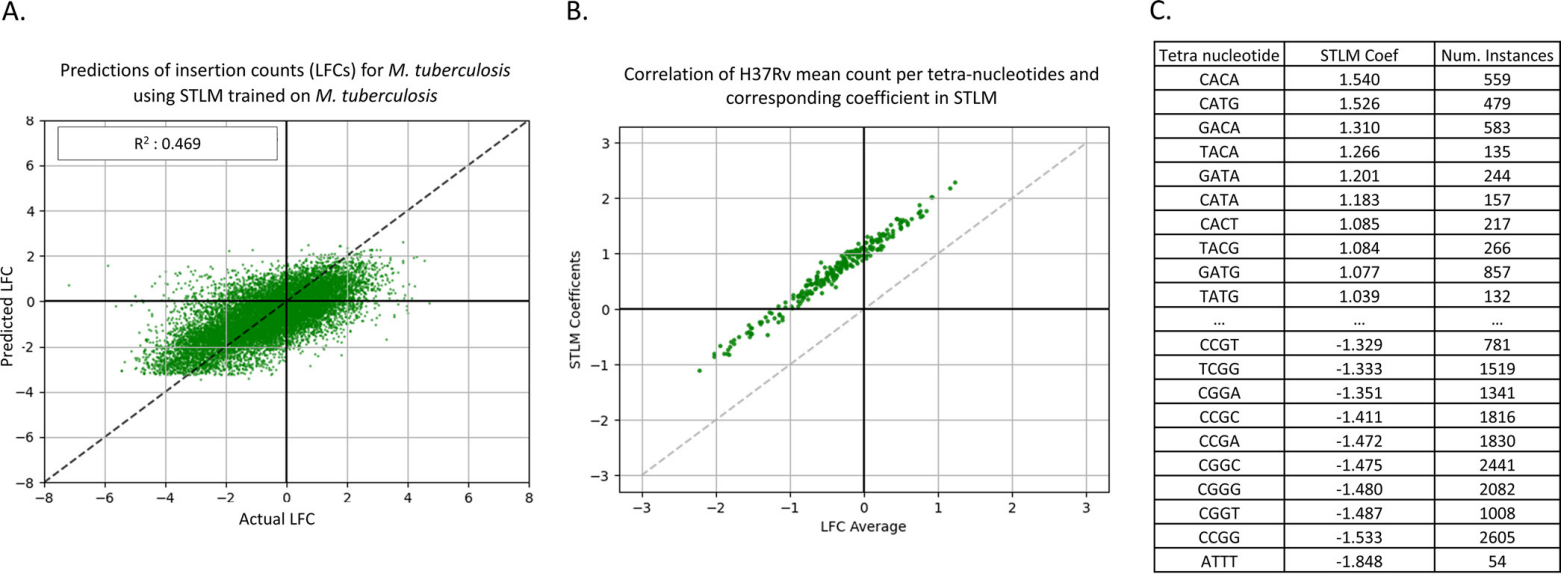
Predicted LFC versus actual LFC using STLM. Panel A shows a plot of the actual LFCs versus the LFCs predicted by our model. The predictive power of this model is about the same as the neural network (*R*^2^ = 0.468), but panel B shows there is a high correlation of mean LFCs of each tetranucleotide and the coefficient in the STLM of the same tetranucleotide, indicating our model represents our data well. Panel C shows the coefficients associated with each tetranucleotide (Table S1), sorted by coefficient value.

As seen in Fig. 6A, 10-fold cross-validation using the H37Rv data resulted in an average *R*^2^ value of 0.469. This *R*^2^ value is slightly lower than, but nearly equal to, that of the neural network (*P* value < 0.01 from two-tailed *t* test). However, the STLM provides us more insight into patterns contributing to the prediction of the LFCs. In a regression with these tetranucleotide features, we expect each coefficient (i.e., weights) of the model to correlate with the average LFC associated with each tetranucleotide (over TA sites surrounded by these tetranucleotides). Figure 6B shows the relationship of the STLM coefficients and the mean observed LFCs of the corresponding tetranucleotides (shifted on the *y* axis by the bias [intercept] in our data). The strong linear trend visible adheres to the expectation of a high correlation and indicates our model accurately represents our data. The individual tetranucleotide coefficients are shown in Fig. 6C, sorted in decreasing order (see Table S1 for full table). Consistent with the patterns observed in the heatmaps in Fig. 4, the bottom 10 features associated with low coefficients (predictive of low mean LFCs) all have a ‘G’ in the second position upstream of the TA sites and a ‘C’ or ‘G’ in the third position. The features associated with the top 10 coefficients, thus higher LFC values, all have an ‘A’ in the third position upstream from the TA site. However, the strength of the STLM is that it accounts for combinations of 4 nucleotides at a time, resolving cases where single-nucleotide patterns might conflict.

10.1128/mSystems.00876-21.8 TABLE S1 Summary of STLM and the tetranucleotides it contains as features. Column A: 4-bp pattern appearing adjacent to TA dinucleotide sites. Column B: STLM coefficient, coefficient associated with the tetranucleotide in the symmetric tetranucleotide linear model. Column C: number of TA sites in H37Rv TnSeq libraries not marked ‘ES’ (essential) in preliminary analysis that contain the tetranucleotide in a window −4 or +4 bp from the TA site (reverse-complemented downstream). Download Table S1, XLSX file, 0.02 MB. Copyright © 2021 Choudhery et al. 2021 Choudhery et al. https://creativecommons.org/licenses/by/4.0/ This content is distributed under the terms of the Creative Commons Attribution 4.0 International license.

While the STLM was able to partially predict the frequency of insertion at different TA sites (*R*^2^ = 0.47), a significant amount of variability remains between observed and predicted insertion counts. This can be attributed to factors that the model did not account for, such as GC content outside the −4…+4 region or DNA bendability (11). However, when the STLM was augmented with the addition of GC content as a feature, where GC content was calculated with the ±20-bp window, it showed an improvement in *R*^2^ of only 0.02. When the STLM was augmented with bendability as an additional feature, calculated for each TA site using the *bend-it* program (12), the results were nearly identical to that of the model with only the 256-bit vectors (*R*^2^ = 0.47). These experiments indicated that the tetranucleotides are a larger factor in the prediction of LFCs than GC content or bendability. Using only the GC content and bendability as two features to a linear model resulted in an *R*^2^ of nearly zero for all the data sets tested. Furthermore, plots of LFC versus bendability and LFC versus GC content showed little to no correlation.

### Application of STLM to other Himar1 TnSeq data sets.

To evaluate whether the nucleotide biases derived from these 14 independent data sets in H37Rv are representative of generalized insertion preferences of the Himar1 transposon, we compared the biases seen so far to those in other Himar1 TnSeq data sets.

**FIG 7:**
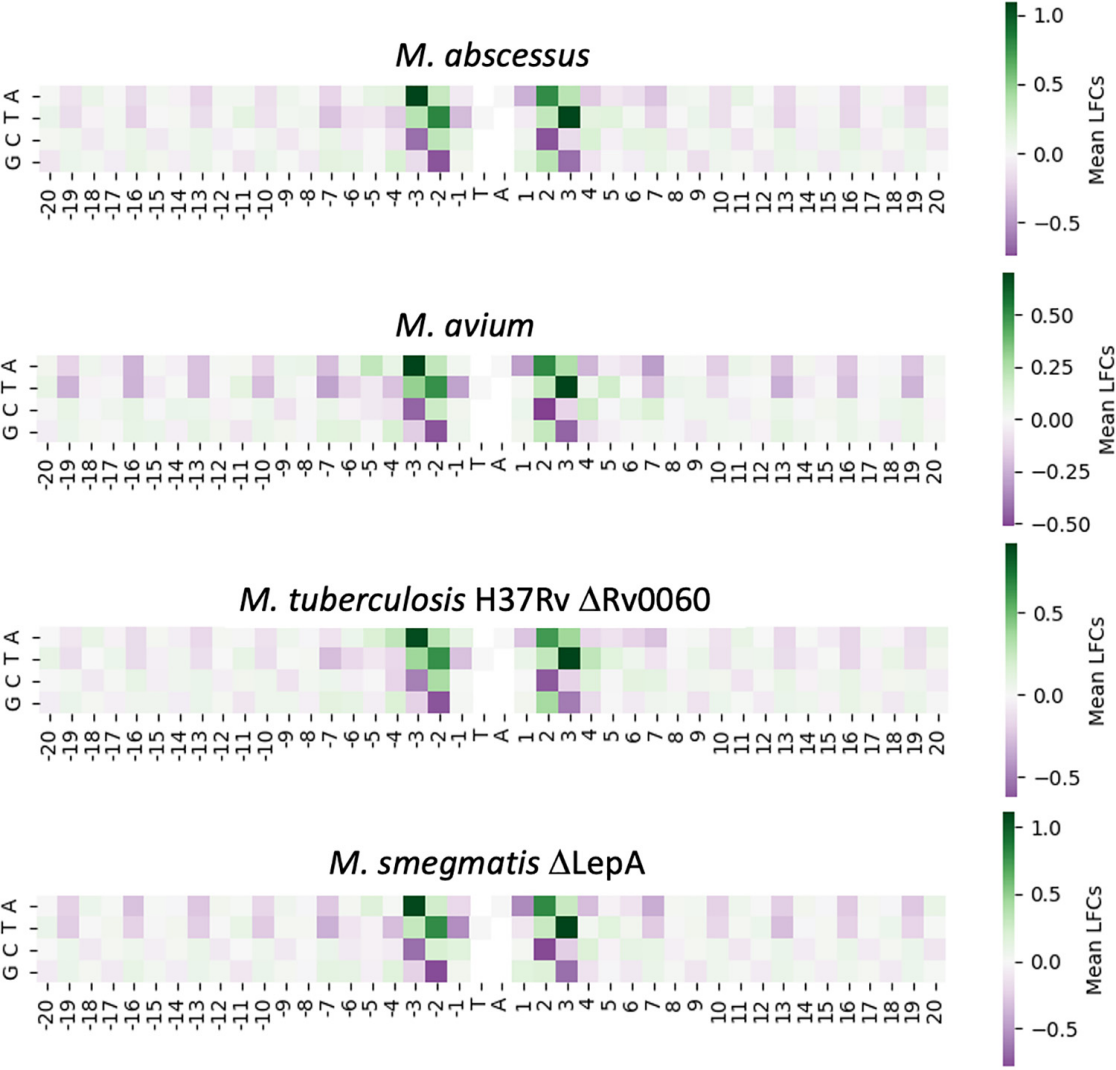
Enrichment and depletion of insertions as a function of nucleotides surrounding TA sites for mycobacterial TnSeq data sets. The four heatmaps are calculated in the same manner that the H37Rv heatmap was calculated in Fig. 4. The mean of each filtered nucleotide at every position in a ±20-bp window around the TA sites is calculated. The patterns of all the heatmaps look very similar to both each other and to the H37Rv heatmap in Fig. 4.

Staying within the Mycobacterium genus, we obtained data sets from Himar1 TnSeq libraries, grown in regular growth medium (7H9), of M. avium (13), M. abscessus ATCC 19977 (14), M. smegmatis mc^2^155 ΔLepA (E. J. Rubin, unpublished data), and M. tuberculosis H37Rv ΔRv0060 (15). We extracted the LFCs from the data sets based on the insertion counts at TA sites in each genome along with tetranucleotide vectors based on the nucleotides surrounding each TA site. The heatmaps for each of the data sets in Fig. 7 show the mean LFCs associated with each nucleotide at each position within a ±20-bp window of the TA site. These heatmaps look nearly identical to the heatmap of H37Rv in Fig. 4. They exhibit the same negative LFC bias for −3 ‘C’, +3 ‘G’, −2 ‘G’, and +2 ‘C’ and the same −3 ‘A’, +3 ‘T’ positive LFC bias. STLM LFC predictions for each of the new TnSeq data sets were adjusted by a simple regression-based procedure to correct for differences in the LFC distribution (further described in Materials and Methods). Results, calculated as correlations between predicted and observed LFCs with the regression adjustment (see Fig. S5), along with the nucleotide biases observed in the heatmaps, show that the STLM can help explain the variability in insertion counts at different TA sites for these data sets (using coefficients trained on M. tuberculosis H37Rv data but applied to data sets from other mycobacterial species). The predictive power of our model on the M. abscessus data set (*R*^2^ of 0.504) is slightly higher than, but about the same as, the M. tuberculosis test set. We ran the same analysis on a recently published TnSeq data set from an independent library of M. abscessus ATCC 19977 (16) as well and observed very similar heatmaps and predictive power (*R*^2^ of 0.477). This shows we can explain ∼50% of the variance in Himar1 insertion counts in this organism based on the nucleotide biases. All the data sets exhibited a correlation between observed and predicted LFCs (and hence insertion counts) and displayed a nucleotide pattern similar to the heatmaps from the other mycobacterial TnSeq data sets.

10.1128/mSystems.00876-21.5 FIG S5 Predictive power of STLM on mycobacterial data sets. The predictive power of the STLM on the mycobacterial data sets has been varied. However, an *R*^2^ value greater than 0.25 for nearly all the data sets indicates that the nucleotide biases explain at least a fourth of the variance in insertion counts with nucleotide biases. Download FIG S5, TIF file, 2.8 MB. Copyright © 2021 Choudhery et al. 2021 Choudhery et al. https://creativecommons.org/licenses/by/4.0/ This content is distributed under the terms of the Creative Commons Attribution 4.0 International license.

**FIG 8:**
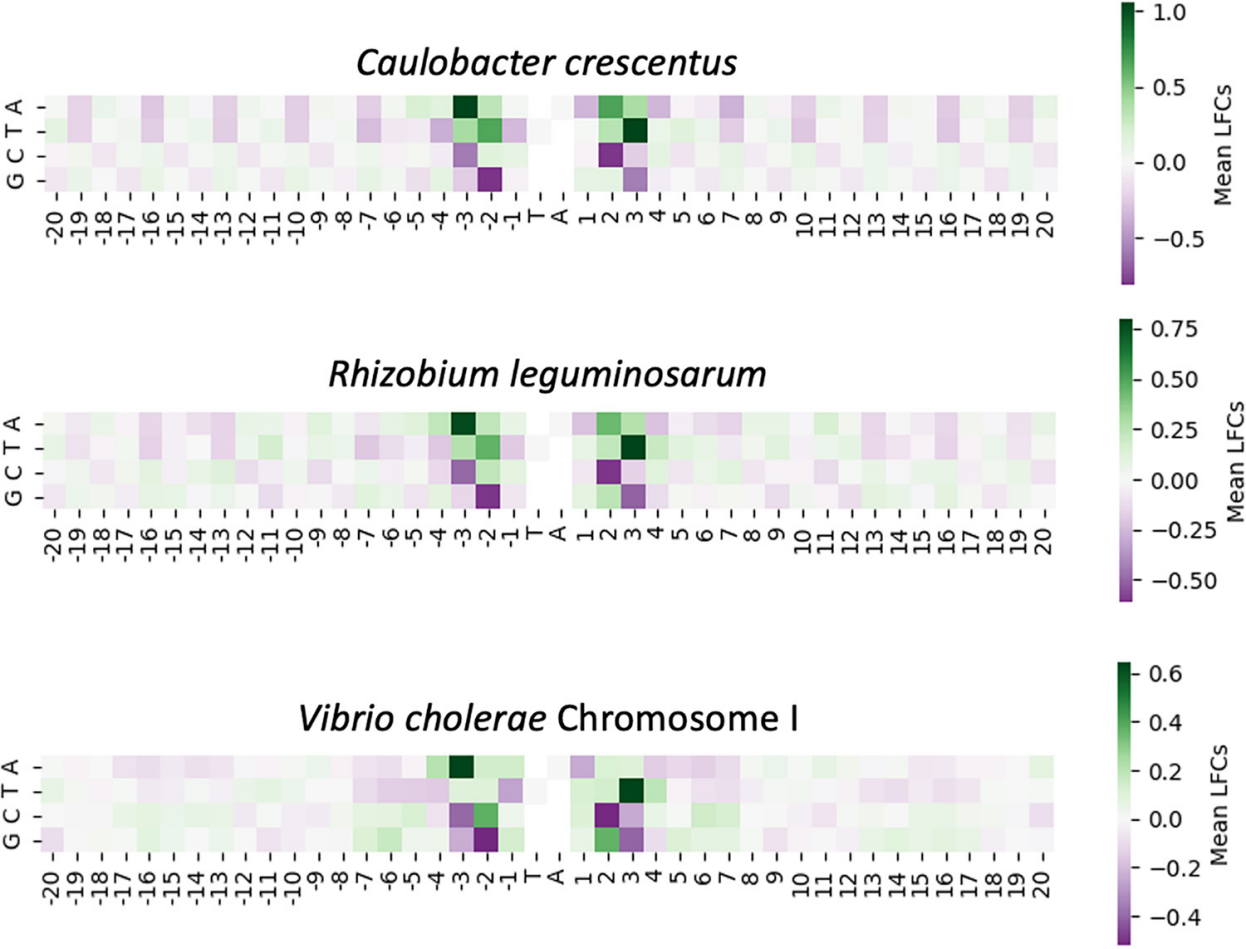
Enrichment and depletion surrounding TA sites for nonmycobacterial TnSeq data sets. The four heatmaps are calculated in the same manner that the H37Rv heatmap (Fig. 4) and mycobacterial heatmaps (Fig. 7) were calculated. The mean of each filtered nucleotide at every position in a ±20-bp window around each TA site is calculated and centered on the median of mean LFCs.

To examine whether these biases also occur outside the Mycobacterium genus, we obtained Himar1 TnSeq data sets from Caulobacter crescentus (17), Rhizobium leguminosarum (18), and Vibrio cholerae (chromosome I only; chromosome II behaves similarly) (19). We calculated LFCs (log fold change of insertion counts relative to local mean) at each TA site in these genomes and plotted the heatmaps as associations of nucleotides at specific positions around the TA with LFCs. As Fig. 8 shows, the heatmaps associated with all three data sets reflect the same nucleotide patterns found in the mycobacterial data sets. Applying the STLM to these data sets yielded significant correlations between predicted and observed LFCs, with statistically significant *R*^2^ values (see Fig. S6). The correlation for Vibrio cholerae is lower than the others (*R*^2^ = 0.249), possibly due to sequence preferences in the fragmentase used for shearing during the sample prep for sequencing. This was done differently than other TnSeq experiments and could have introduced additional variance into the insertion counts for the *Vibrio* data set. However, the heatmap shows a pattern consistent with the nucleotide biases we see with the TnSeq data sets from other organisms. This indicates that the nucleotide biases visible in the mycobacterial data sets also explain some of the insertion count variances present in nonmycobacterial data sets, thus supporting that the STLM captures generalized site-specific biases on insertion preferences of the Himar1 transposon.

10.1128/mSystems.00876-21.6 FIG S6 Predictive power of STLM on nonmycobacterial data sets. The predictive power of the STLM on nonmycobacterial data sets has been more varied than the mycobacterial data sets. *Caulobacter* has a high *R*^2^ value, whereas *Vibrio* has quite a low *R*^2^ value. However, an *R*^2^ value greater than 0.10 for nearly all the data sets indicates that the nucleotide biases explain at least some of the variance in insertion counts with nucleotide biases. Download FIG S6, TIF file, 2.6 MB. Copyright © 2021 Choudhery et al. 2021 Choudhery et al. https://creativecommons.org/licenses/by/4.0/ This content is distributed under the terms of the Creative Commons Attribution 4.0 International license.

### SNPs around TA sites in an M. abscessus clinical isolate exhibit predictable changes in insertion counts.

**FIG 9:**
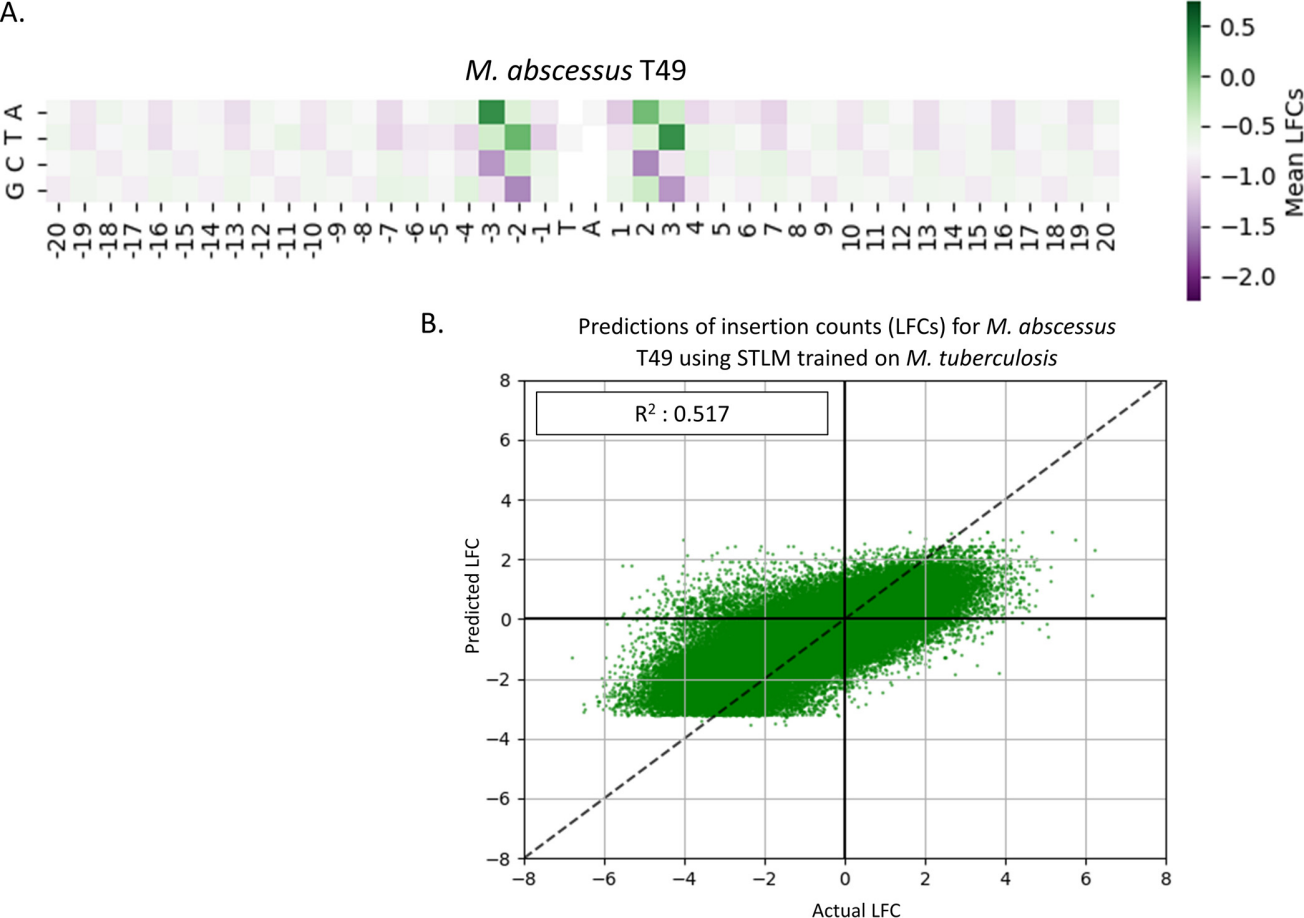
M. abscessus Taiwan49 clinical isolate data set. The heatmap in panel A is calculated in the same manner that the previous heatmaps were calculated. The pattern of this heatmap looks very similar to the H37Rv heatmap (Fig. 4) as well as the heatmap for the M. abscessus ATCC 19977 reference strain (Fig. 8). The predictive power of the STLM on the M. abscessus T49 data set in panel B shows a high *R*^2^ value of 0.517, like that of the M. abscessus reference data set. This indicates that nucleotide biases explain at least half of the variance in insertion counts for this data set with nucleotide biases.

To evaluate whether changes in nucleotides proximal to TA sites would have a predictable effect on transposon insertion counts, we obtained a Himar1 Tn library for a clinical isolate of M. abscessus Taiwan49 (M. abscessus T49) and compared it to a Tn library in the reference strain, ATCC 19977 (generated by methods described in the work of Akusobi et al. [14]; see “Data availability” for data files with raw insertion counts). These two strains of M. abscessus are fairly divergent, belonging to different subspecies (ATCC 19977 in Mycobacterium abscessus subsp. *abscessus*, and Taiwan49 in Mycobacterium abscessus subsp. *massiliense*); they have 114,335 single nucleotide polymorphisms (SNPs) between them based on a genome-wide alignment. However, at the level of functional genomics, they are similar. As determined through the HMM method in TRANSIT (9), 513 out of 4,923 total genes in ATCC 19977 and 451 out of 4,225 total genes in T49 are predicted to be essential or growth defect genes. Four hundred seventeen of these genes overlap. Figure 9 shows that predicting insertion counts in this isolate with the STLM yielded an *R*^2^ value of 0.49. This is, as expected, quite similar to the results of the M. abscessus reference data set reported above. After aligning the genomes of the M. abscessus Taiwan49 clinical isolate and the M. abscessus reference strain, we found 9,303 TA sites where there was exactly one SNP in the 8-nucleotide window (±4 bp) surrounding the TA site.

**FIG 10:**
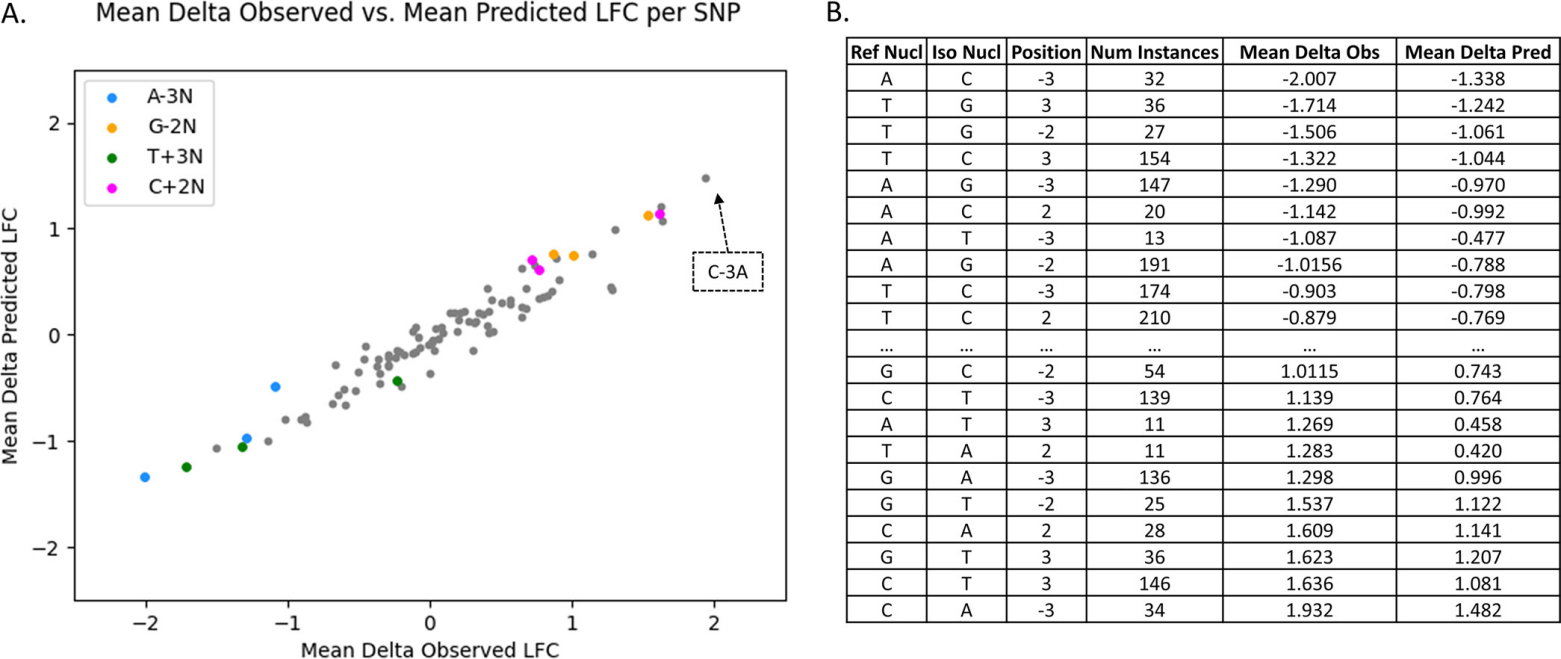
SNPs in M. abscessus Taiwan49 clinical isolate exhibit predictable changes in nucleotide biases. Panel A shows the correlation of changes in observed versus predicted LFCs for the 96 possible SNPs in the ±4-bp window around the TA site (taking reverse-complement for those downstream). The colored markers are the nucleotide-position pairs previously found to have the highest biases. The table in panel B is sorted by increasing mean delta observed LFC and provides more details on these SNPs. As expected, the most extreme changes occur when the SNP occurs in the −3, −2, +2, or +3 position. The top 10 and bottom 10 values, i.e., the biggest decreases and biggest increases in LFC, follow the heatmap patterns of the Himar1 data sets tested.

A plot of the average changes in observed LFCs versus the average changes in predicted LFCs between the reference and isolate strain at these sites for every TA site with an adjacent SNP can be seen in Fig. 10 (see Materials and Methods). We expected that when a nucleotide with a high negative bias was mutated, the observed LFC would increase, and when a nucleotide with a high positive bias was changed, the observed LFC would decrease. Figure 10 shows this effect. The colored points in the graph are the most significant nucleotide-position pairs that we have previously observed to have the highest LFC biases. When a nucleotide is switched from an ‘A’ in the −3 position (blue) or a ‘T’ in the +3 position (green) to any other nucleotide, there is a decrease in observed LFC, and when a ‘G’ in the −2 position (orange) or ‘C’ in the +2 position (pink) is changed, there is an observed increase. The presence of this effect of SNPs on the LFC, i.e., differences in insertion counts at corresponding TA sites in different clinical isolates, along with the high correlation of the observed and predicted LFC changes, provides further evidence that the STLM can represent the nucleotide biases on transposon insertion preferences with high accuracy.

The accompanying table in Fig. 10 is a truncated view of the SNPs sorted in increasing order of mean observed LFC change (for the full table, see Table S2). In addition to the general pattern observed in the plot, we see that the magnitudes of the LFC differences correspond to the magnitudes of nucleotide biases. In the previous heatmaps, ‘A’ in the −3 position (and the downstream reverse-complemented pattern) shows the strongest bias for high LFCs and ‘C’ in the −3 position or ‘G’ in the −2 position (and the downstream reverse-complemented pattern) shows the strongest bias toward low LFCs. Following this pattern, the biggest decrease in mean observed LFC occurred when an ‘A’ in the −3 position was changed to a ‘C’ and the biggest increase in mean observed LFCs occurred when ‘C’ in the −3 position was changed to an ‘A’. Thus, the effect of SNPs between a pair of moderately divergent strains corresponds to the nucleotide biases observed within other Himar1 TnSeq data sets and furthers the notion that these biases are general and can explain a significant portion of the variance in transposon insertion counts.

10.1128/mSystems.00876-21.9 TABLE S2 Summary of TA sites with exactly one proximal SNP in a whole-genome alignment between the reference strain (ATCC 19977) and clinical isolate T49 of M. abscessus. Reference nucleotide: nucleotide at position from the TA site mentioned in the reference strain (ATCC 19977). Isolate nucleotide: nucleotide at position from the TA site mentioned in the clinical isolate strain M. abscessus T49. Number of instances, number of times that difference between nucleotide in reference and nucleotide in isolate occurs at position from the TA site. Mean delta observed LFC, mean observed LFC of TA sites in the reference strain with the reference nucleotides at position mentioned − mean observed LFC of TA sites in the reference strain with the isolate nucleotides at position mentioned. Mean delta observed LFC, mean predicted LFC of TA sites in the reference strain with the reference nucleotides at position mentioned − mean predicted LFC of TA sites in the reference strain with the isolate nucleotides at position mentioned. Download Table S2, XLSX file, 0.01 MB. Copyright © 2021 Choudhery et al. 2021 Choudhery et al. https://creativecommons.org/licenses/by/4.0/ This content is distributed under the terms of the Creative Commons Attribution 4.0 International license.

### Nucleotide biases observed in insertions at TA sites in a Tn*5* TnSeq data set are different than the biases that occur in Himar1 TnSeq data sets.

To evaluate whether the patterns of bias we see in the Himar1 TnSeq data sets are due to biases of the transposase itself, and not due to the process of generating the libraries (including cellular factors such as cognate DNA-binding proteins that might influence transposon insertion during transfection in living cells, for example), we analyzed a Tn*5* TnSeq library of Salmonella enterica serovar Typhi Ty2. Though Tn*5* is from a different (non-*mariner*) family of transposases (20), the Tn library was generated and sequenced using a similar methodology (PCR amplification of transposon insertion junctions, followed by short-read sequencing on an Illumina sequencer) (21). We analyzed the Salmonella baseline (*in vitro*) data set similarly to the way we analyzed the Himar1 data sets, except we restricted attention to TA sites with insertion counts greater than 0 (22,406 of 230,942 TA sites had insertions) and used the global nonzero mean at TA sites as a reference to calculate LFCs. Although the logo plot in Fig. S7 shows biases present for Tn*5* insertion at TA sites, they clearly differ from the biases observed for Himar1 insertion. Starting at position −10 from the TA site, we see a preference for the consensus sequence AGTWTWAGACT, where W stands for A or T. This is similar to the published weak consensus pattern (from a much smaller data set) for Tn*5* preferred target sites: “AGNTYWRANCT” (22). Like the sequence biases observed with Himar1, the pattern for Tn*5* can also be seen reverse-complemented downstream of the TA site. Since TA sites in the Tn*5* and Himar1 TnSeq data sets reveal completely different nucleotide patterns, we infer that the biases observed in Himar1 libraries are not due to the process of constructing or sequencing the library but are, rather, specific to the Himar1 transposase itself.

10.1128/mSystems.00876-21.7 FIG S7 Enrichment and depletion of insertions as a function of nucleotides surrounding TA sites for Salmonella enterica serovar Typhi Ty2 Tn*5* TnSeq data set. The logoplot is calculated in the same manner that the heatmaps for the Himar1 data sets were calculated in Fig. 4, 7, and 8. The mean LFC (LFC = log_2_ of counts of sites with that nucleotide in that position relative to the genome-wide average count at nonzero TA sites) of each filtered nucleotide at every position in a ±20-bp window around the TA sites is calculated. Nucleotides above the line are associated with sites with higher-than-average counts, and nucleotides below the line are associated with sites with lower-than-average counts. The box indicates the region upstream of the TA site that matches the previously published consensus pattern for preferred Tn*5* insertion sites. Download FIG S7, TIF file, 2.9 MB. Copyright © 2021 Choudhery et al. 2021 Choudhery et al. https://creativecommons.org/licenses/by/4.0/ This content is distributed under the terms of the Creative Commons Attribution 4.0 International license.

### Using expected insertion counts to improve gene essentiality predictions.

Previous methods of identifying essential genes within individual data sets have been based on the magnitude of insertion counts. For example, tools such as TnSeq-Explorer (23) use the mean of insertion counts in sliding windows to classify genes by essentiality. The limitation of relying on raw insertion counts is that they can be highly variable among TA sites, and this noise can lead to inaccurate estimation of the relative level of fitness defects caused by transposon disruption. We describe a new method, called the TTN-Fitness method using the Gene+TTN model, which considers the site-specific biases on Himar1 insertion preferences to correct the observed counts for expectations based on the nucleotides surrounding each site.

The Gene+TTN model incorporates nucleotide context into an insertion count-based model, allowing us to decouple the two main causes for low insertion counts: biological and Himar1 insertion preferences. This allows us to make a more informed assessment on the level of gene fitness defect for biological reasons. The input to the model for each TA site is a vector consisting of a binary encoding of the gene in which it is located, combined with the 256 tetranucleotide (TTN) features. Each TA site is represented as a bit vector, with 3,981 features, one for each gene, and 256 features encoding the upstream and reverse-complemented downstream tetranucleotides adjacent to the site. We excluded TA sites from genes determined to be ‘Essential’ through the Gumbel analysis (24) and Binomial Distribution (see Materials and Methods), which quantify the significance of gaps (or runs of consecutive TA sites lacking insertions) to identify essential regions. The model can be represented in matrix form as

(M_1_) Y=b + CG + DT

where *Y* is a vector of the log_10_ of insertion counts at every TA site, *G* is the matrix of 3,981 gene covariates for each site, *C* is vector coefficients to be fit per gene, *T* is the matrix of 256 tetranucleotide covariates for each site, and *D* is the vector of coefficients to be fit per tetranucleotide. The intercept *b* is close to the global average of log_10_ insertion counts, and the coefficients (*C*) for every gene reflect the deviation of the gene’s mean log_10_ insertion count from the global average, adjusting for the effect of surrounding nucleotides (*D*). Essentially, we are finding the deviation of the gene’s mean insertion count from the global average based on biological reasons, i.e., subtracting out the effect of site-specific nucleotide-based Himar1 insertion preferences. Thus, the gene-specific coefficients (*C*) represent adjusted estimates of the fitness level of each gene.

**FIG 11:**
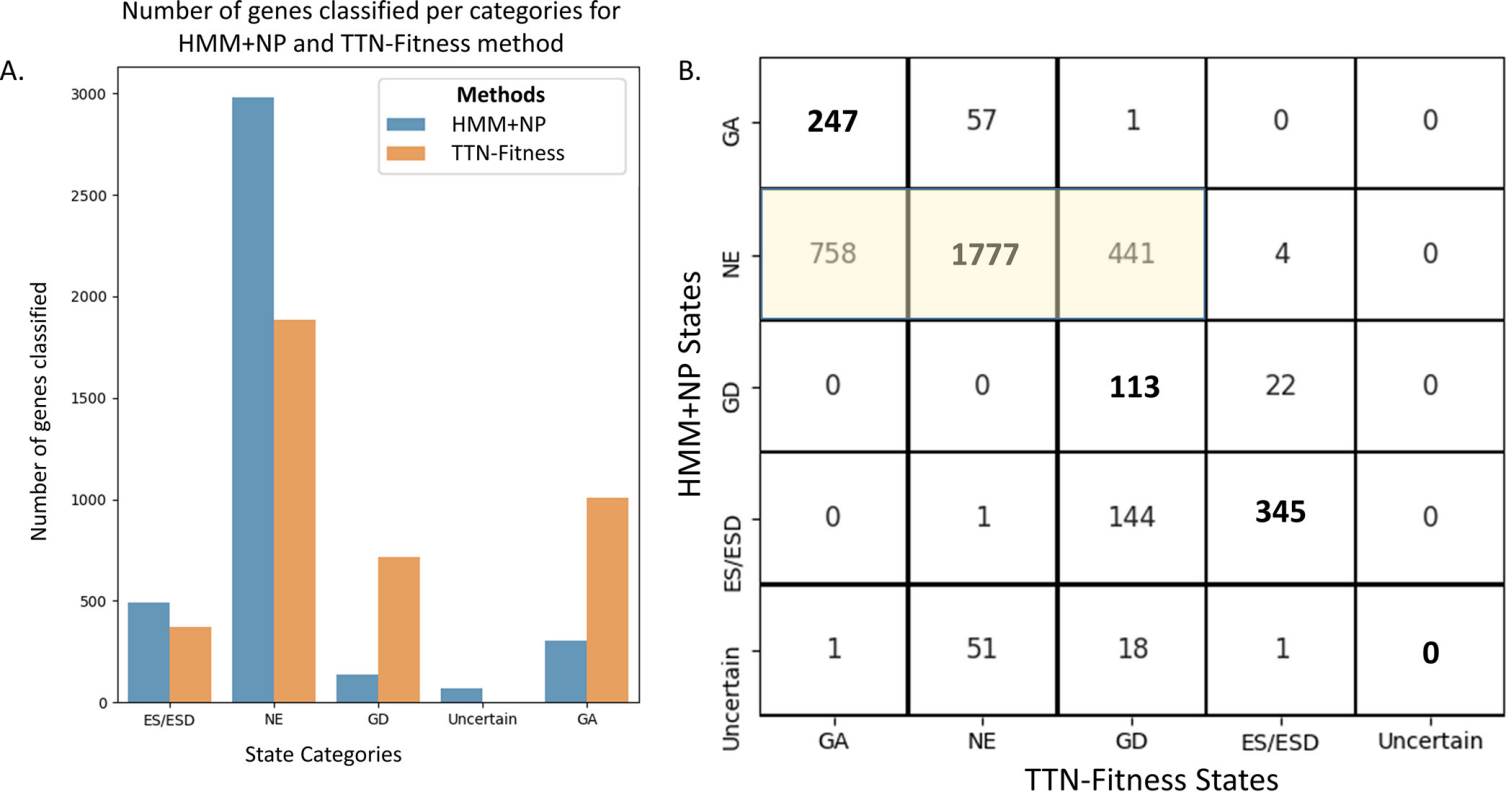
Distribution of HMM+NP and TTN-Fitness states for genes provided in the M. tuberculosis H37Rv data set. Panel A shows the distribution of classification of genes by the two methods. Panel B shows the confusion matrix of the classification of genes in the two methodologies. Most of the genes are labeled NE in both models. Genes determined to be ‘Uncertain’ in the HMM+NP model are assigned other states in the TTN-Fitness method. A fraction of genes labeled ‘NE’ (Non-Essential) in the HMM+NP model (highlighted matrix components) are reassigned as ‘GA’ (Growth Advantage) or ‘GD’ (Growth Defect) using the TTN-Fitness method, indicating that the TTN-Fitness method is more sensitive in estimating fitness than the HMM+NP model.

The regression model was trained on the M. tuberculosis H37Rv *in vitro* data set. The significance of genes (i.e., *P* value) was calculated using a *t* test (25) and then adjusted for multiple testing to limit the false-discovery rate (FDR) to ≤5% using the Benjamini-Hochberg method (26). Genes with an adjusted *P* value of <0.05 and negative coefficient are interpreted as ‘Growth Defect’ (GD) genes, and those with an adjusted *P* value of <0.05 and a positive coefficient are interpreted as ‘Growth Advantaged’ (GA) genes. Genes with an insignificant coefficient near 0 (adjusted *P* value > 0.05) are interpreted as ‘Non-Essential’ (NE). Genes identified *a priori* as essential by the Gumbel method in TRANSIT (9) were marked ‘Essential’ (ES) by the TTN-Fitness method and excluded from both training and testing. Gumbel identifies large essential genes well but tends to classify small genes (with <10 TA sites) as ‘Uncertain’, depending on the overall level of saturation of the data set. Thus, we use the binomial distribution to classify additional significant genes (*P* < 0.05) lacking insertions that are likely essential as ‘Essential-B’ (ESB, as a subcategory of ES) (see Materials and Methods). The HMM+NP model, a modified HMM to account for nonpermissive sites described by DeJesus et al. (8), distinguishes between ‘ES’ and ‘ESD’ (Domain-Essential) genes, which our model does not. For model comparison, we have combined the two categories into one labeled ‘ES/ESD’. As seen in Fig. 11A, the TTN-Fitness method labels a similar number of genes essential as the HMM-NP method, though slightly fewer nonessential and more in the growth-defect and growth-advantaged categories (8). The confusion matrix in Fig. 11B shows that there are 345 genes labeled ‘Essential’ in both the TTN-Fitness method and the HMM+NP model (i.e., on the diagonal in the confusion matrix), showing a great deal of overlap. Between the 2 methods, 1,777 ‘Non-Essential’ genes also overlap. However, the biggest difference is that a large number of genes labeled ‘Non-Essential’ (NE) by the HMM get reclassified as either ‘GD’ or ‘GA’ by the TTN-Fitness method. Of genes labeled ‘Non-Essential’ in the HMM+NP model, 14.7% have slightly lower than average insertion counts and are classified as ‘GD’ via the Gene+TTN (M_1_) model. Of genes labeled ‘Non-Essential’ by the HMM+NP model, 25.4% have insertion counts slightly higher than average and are classified as ‘GA’ through the Gene+TTN (M_1_) model. This shows that the TTN-Fitness method labels genes similarly to the HMM+NP model for the most part but is more sensitive to deviations from the average insertion count and consequently labels some genes more specifically as ‘GD’ or ‘GA’.

**FIG 12:**
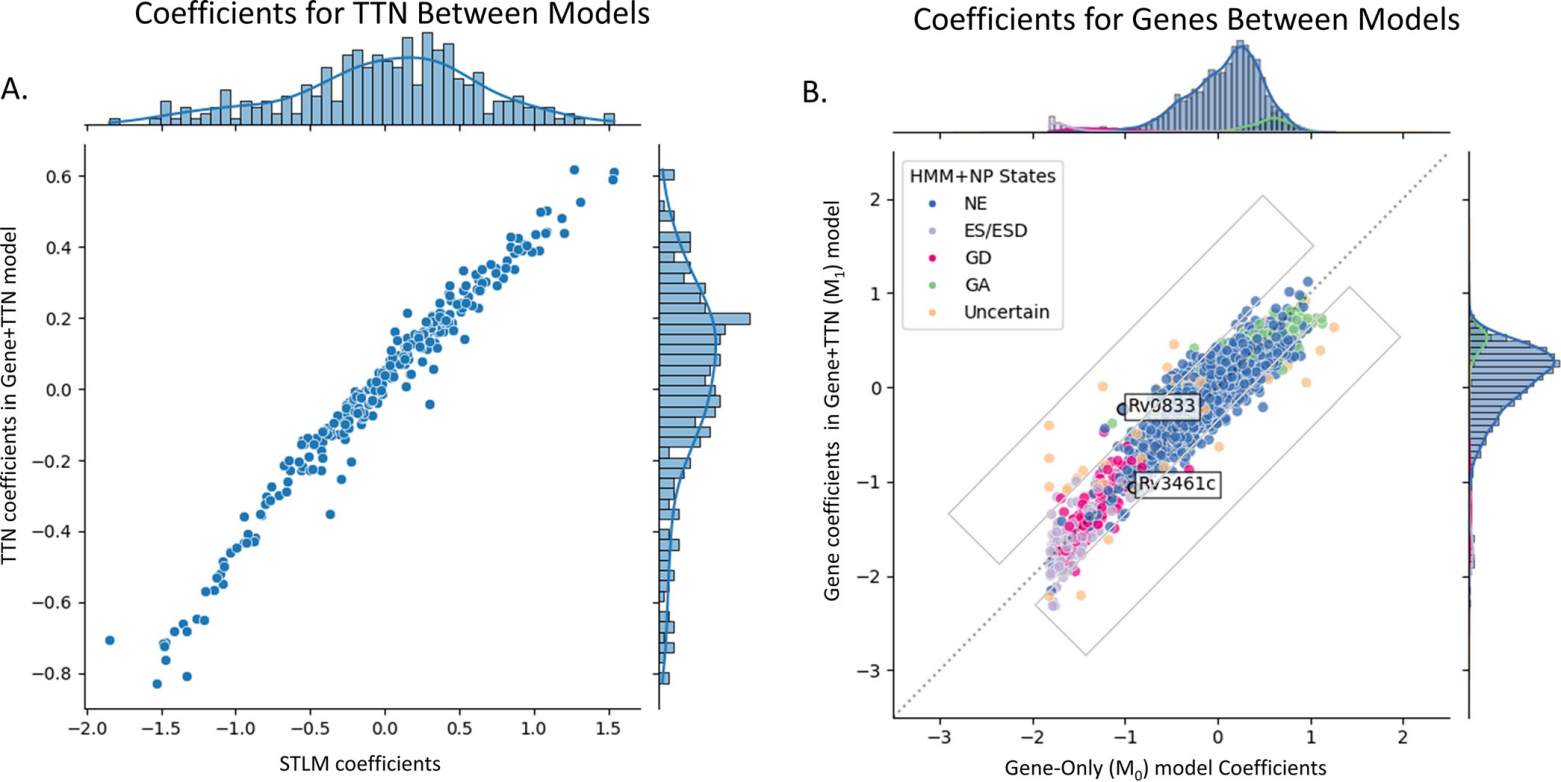
Correlation of coefficients in the Gene+TTN model (of the TTN-Fitness method) and coefficients in models using its components. Correlation of coefficients of TTNs in the STLM and the TTN-Fitness model (A) has a strong linear relationship as well as similar distributions, indicating that the models incorporate the effects of TTNs on the insertion count in the same way. Correlation of gene coefficients between the Gene-Only model and the TTN-Fitness model (B) shows a linear trend, indicating that most genes behave in the same way and yield similar results in the two models. However, there are a few that are show log fold change greater than this majority. The scale of coefficients in the Gene+TTN model is greater than that in the Gene-Only model, indicating a notable number of genes whose predicted fitness estimate changes with the inclusion of nucleotide context. The points with black outlines and labels are genes that we have explored. The gray boxes above and below the *y* = *x* line in the plot are the regions where the top 20 and bottom 20 genes lie, respectively.

Figure 12A shows a linear relationship between the coefficients associated with tetranucleotide features in the Gene+TTN (M_1_) model and the corresponding coefficients of the STLM, illustrating that the influence of tetranucleotides on predicted counts captured in this model is consistent with the effect previously discussed in the STLM. Figure 12B shows the difference in the fitness assessment of genes compared to a Gene-Only (M_0_) model, dropping the TTN features and hence lacking the site-specific adjustments based on tetranucleotide covariates. The Gene-Only (M_0_) model encodes only the gene at every TA site and can be expressed in matrix form as

(M_0_) Y=b + CG

The intercept (*b*) is the global average log_10_ insertion count in the genome, and the coefficient *C* corresponding to each gene is the deviation of the gene’s mean insertion count from the global average. As this model does not incorporate the tetranucleotides, if there is a gene with a very negative coefficient, it will be interpreted as ‘Growth Defect’ regardless of whether the suppression of insertions is due to a true biological gene defect or nucleotide bias. The scatterplot of the gene coefficients between the two models in Fig. 12A shows a strong linear trend, indicating that estimated mean (log_10_) insertion counts for most genes are quite similar between the two models. However, the dispersion suggests that taking the nucleotide context into account changes the fitness estimate for a number of outlying genes. Genes that show the highest differences in coefficients between the two models are frequently labeled ‘Uncertain’ by the HMM+NP model (8), a majority of which are small genes with fewer than 5 TA sites. Details on the difference in the coefficients and their significance (determined through a Student *t* test and an FDR-adjusted *P* value) can be found in Table S3.

10.1128/mSystems.00876-21.10 TABLE S3 Summary of fitness assessment with various methods, including TTN-Fitness. Columns A to F give basic information about each gene in the M. tuberculosis H37Rv genome. Columns H, I, M, and R refer to a reduced linear model, where essentiality of each gene is based only on the average insertion count. Columns J, K, M, and R refer to the TTN-Fitness model, where the nucleotide context of each TA site is taken into account to estimate the expected count at each site. Columns P and Q give essentiality calls by prior methods (Gumbel; M. A. DeJesus, Y. J. Zhang, C. M. Sassetti, E. J. Rubin, et al., Bioinformatics 29:695–703, 2013, https://doi.org/10.1093/bioinformatics/btt043) and a hidden Markov model (HMM+NP; M. A. DeJesus, E. R. Gerrick, W. Xu, S. W. Park, et al., mBio 8:e02133-16, 2017, https://doi.org/10.1128/mBio.02133-16). Column S gives the essentiality call by the TTN-Fitness models (NE, nonessential; GD, growth defect; ES/ESB, essential; GA, growth advantage). Download Table S3, XLSX file, 0.7 MB. Copyright © 2021 Choudhery et al. 2021 Choudhery et al. https://creativecommons.org/licenses/by/4.0/ This content is distributed under the terms of the Creative Commons Attribution 4.0 International license.

To identify genes with the biggest differences in predicted counts in the Genes+TTN model (M_1_) versus the Gene-Only model (M_0_), we extracted the coefficients for each gene from both models and sorted the genes based on the difference of the two coefficients (Table S3). The coefficient difference for each gene was calculated as the M_0_ coefficient subtracted from the M_1_ coefficient for that gene. In Table 1, we provide a list of the top 20 and bottom 20 genes (filtering out genes with less than 1 TA site with insertions) for which the TTN features make the biggest difference, along with a summary of some statistics for each group and the effect on essentiality categorizations. The category of genes with the greatest increase in coefficient value due to the incorporation of surrounding nucleotides (“Top 20”) have a higher average GC content than the average gene in the genome. A higher GC content means a higher possibility that a TA site in these genes follows the nonpermissiveness pattern (contains a ‘G’ in the −2 position, a ‘C’ in the +2 position, a ‘C’ in the −3 position, or a ‘G’ in the +3 position from the TA site). As expected, these genes have a lower-than-average observed insertion count. M_1_ predicts insertion counts for these genes with greater accuracy than M_0_. It reclassifies 7 genes (including 4 of the 5 PGRS genes in the Top 20), which were labeled ‘GD’ by M_0_ based on just the average insertion count in each gene, as ‘NE’ with the incorporation of the nucleotide biases. In addition, M_0_ underpredicts insertion counts for the Bottom 20 genes and, as a result, classifies 5 of these genes as ‘GA’. M_1_ considers the nucleotide context, including an ‘A’ in position −3 or a ‘T’ in position +3, and predicts insertion counts much closer to the actual observed counts and classifies only 1 gene as ‘GA’. Thus, the inclusion of surrounding nucleotide context into the model improves the accuracy of predicting expected counts and, ultimately, the assessment of gene fitness.

**TABLE 1:**
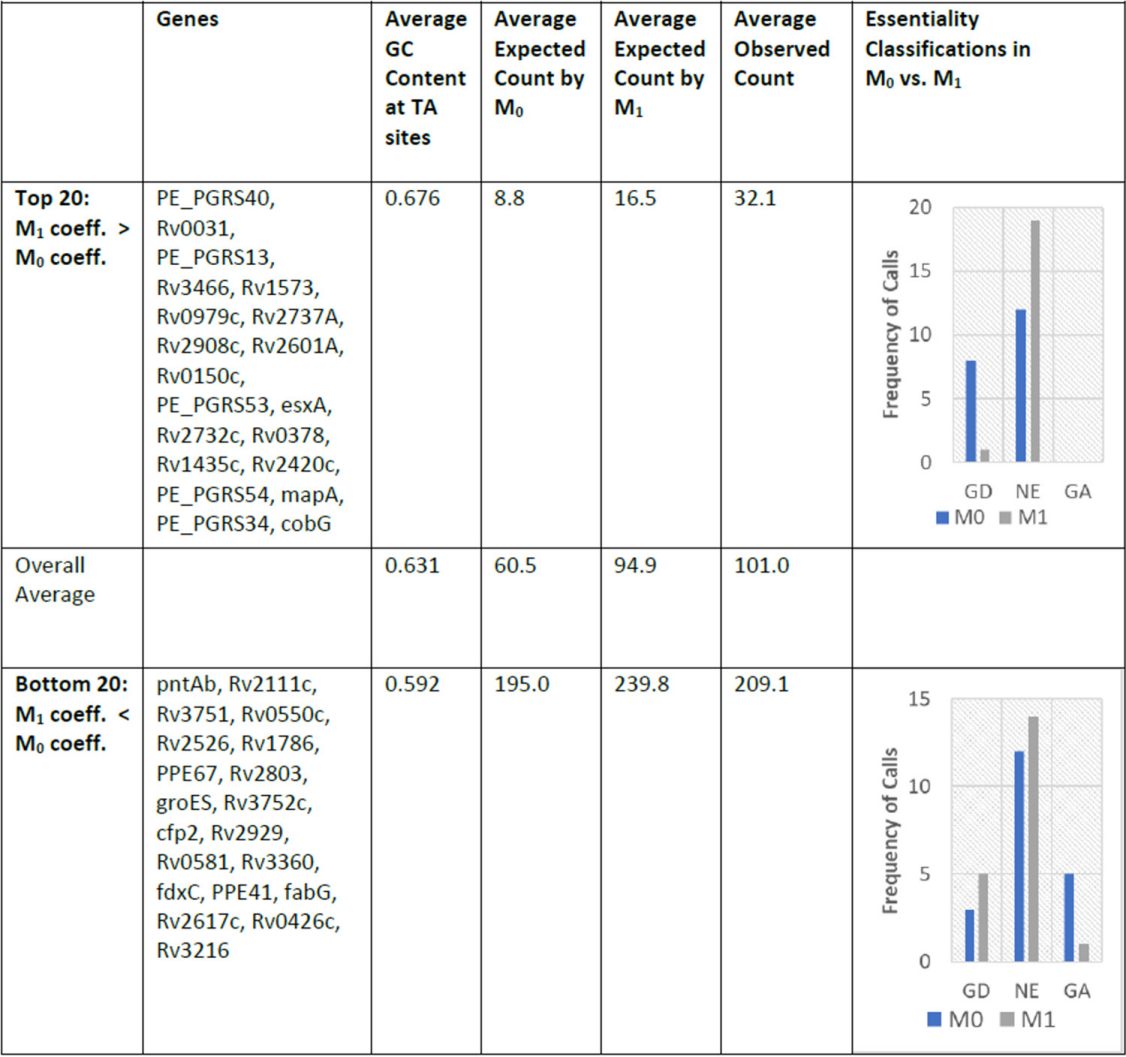
Changes in fitness assessment with the incorporation of surrounding tetranucleotides^*a*^

a The table shows a comparison of three categories of genes, with one where the coefficient in M_1_ (Gene+TTN features) is higher than the coefficient in M_0_ (Gene-Only features) and one where the coefficient in M_1_ is lower than the coefficient in M_0_. The relationship between the GC content and observed count (averaged over the TA sites in each gene) reflects the proposed insertion count biases. The insertion count expected by M_1_ (taking surrounding nucleotides into account) is closer to the average observed count than the insertion count expected by M_0_ (looking only at insertion counts) for all the categories listed. The bar charts in the right column demonstrate that incorporating nucleotide biases, as is done in the TTN-Fitness method, changes essentiality calls from GD (Growth Defect) to NE (Non-Essential) for many of the top 20 genes, and from GA (Growth Advantaged) to NE for several in the bottom 20.

As an example, Rv0833 (PE_PGRS13) is one of the genes in the Top 20 genes whose fitness interpretation is changed from ‘GD’ to ‘NE’ via the Gene+TTN model in the TTN-Fitness method (compared to Gene-Only model). This change in essentiality call is consistent with the fact that PGRS13, like all other PE_PGRS genes, was found to be nonessential *in vitro* in reference 8, and genes in the PGRS family are not known to have a critical function *in vitro* (27). As seen in Fig. 12B, it is interpreted as ‘Growth Defect’ through model M_0_ (Gene-Only; coefficient = −1.02, adjusted *P* value = 2.95 × 10^−6^) and as ‘Non-Essential’ by model M_1_ (Gene+TTN; *C* = −0.26, adjusted *P value* = 0.109, hence not significantly different from 0). The difference in labeling indicates that, based on the surrounding nucleotides, the low insertion counts at TA sites in Rv0833 are predictable. This is supported by the fact that the PE_PGRS genes are especially GC rich (27). The gene contains 12 TA sites spanning 2,250 bp. Of the nucleotides in the ORF, 81.3% were ‘G’s or ‘C’s and 6 sites contained the nonpermissiveness pattern. Thus, observed insertion counts in the gene are much lower than the global average insertion count, but they are expected to be. PE_PGRS13 is one of the 53 PE_PGRS genes (out of 62 total PE_PGRS genes annotated in the M. tuberculosis genome) labeled ‘NE’ by both the HMM+NP and Gumbel methods. Thus, the TTN-Fitness method (incorporating nucleotide context via the Gene+TTN model features) was able to correctly evaluate the fitness of PE_PGRS13 as nonessential, consistent with our understanding of its lack of known function *in vitro*.

**FIG 13:**
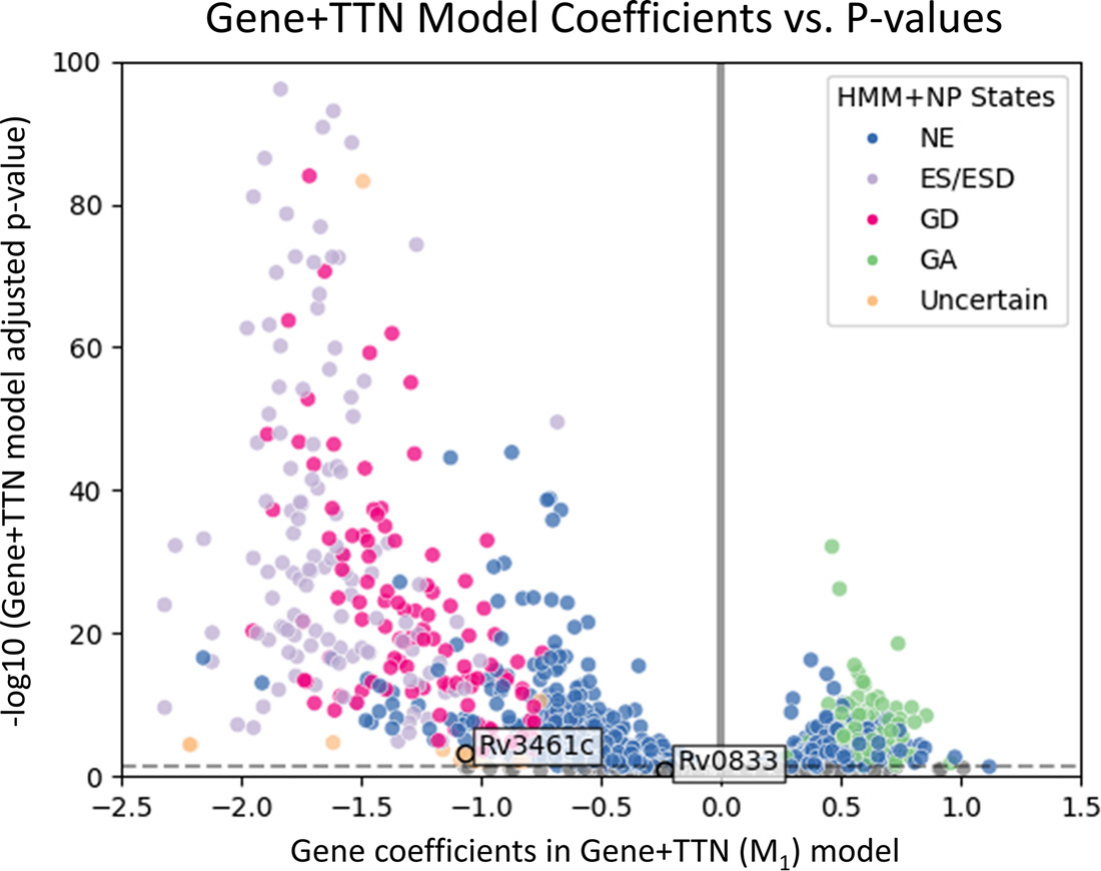
Plots of gene coefficients versus adjusted *P* values in the Gene+TTN model, colored by states determined by the HMM+NP model. The HMM+NP methodology labels genes as ‘Non-Essential’ (NE), ‘Essential’ (ES/ESD), ‘Growth Defect’ (GD), ‘Growth Advantage’ (GA), and ‘Uncertain’. ‘Uncertain’ genes are typically smaller genes. The horizontal dashed line is where adjusted *P* value is 0.05 in the Gene+TTN model. By the TTN-Fitness method, genes below that line are insignificant (gray) and thus ‘NE’. The vertical solid line is where gene coefficient C = 0 in the Gene+TTN model. By the TTN-Fitness method, colored points left of the line are ‘GD’ genes and colored points to the right are ‘GA’ genes. The genes with labels are discussed in the text.

To investigate genes that exhibit large differences in fitness assessment between the TTN-Fitness method and the HMM+NP method, Fig. 13 shows a volcano plot of the gene coefficients from the Gene+TTN model versus the −log_10_ of the FDR-adjusted *P* value. The gray points in the plot are gene coefficients that were not seen to significantly deviate from 0. These are interpreted as ‘nonessential’ genes by the TTN-Fitness method. The genes that were found to be significant are colored according to their labels in the HMM+NP model. The vertical solid line at *C* = 0 is where the colored genes on the left are interpreted as ‘GD’ and colored genes on the right are interpreted as ‘GA’ by the TTN-Fitness method. All significant genes labeled ‘GA’ or ‘GD’ by the HMM+NP model fall on their respective sides of the *C* = 0 line, but there are a few ‘nonessential’ and ‘Uncertain’ genes that are reclassified by the TTN-Fitness method.

With improvements in fitness assessment from the incorporation of tetranucleotides, small genes (3 or fewer TA sites) labeled ‘Uncertain’ by the HMM+NP model can be evaluated with greater confidence. Of the 71 genes labeled ‘Uncertain’ by the HMM+NP model, most (65) have 3 or fewer TA sites, indicating the uncertainty comes from the short length of the gene. These genes are all concretely classified by the TTN-Fitness method (mostly as ‘Non-Essential’ [51 genes] or ‘Growth-Defect’ [18 genes]) (Fig. 11B). Rv3461c (*rpmJ*, 50S ribosomal protein L36), a gene with 3 TA sites, is an example of such an ‘Uncertain’ gene (8). The gene is seen in Fig. 12B to be interpreted as ‘Non-Essential’ by the Gene-Only model M_0_ (*C* = −0.87, adjusted *P* value = 0.074, not significantly different from 0) and ‘Growth Defect’ by the Gene+TTN (M_1_) model (*C* = −1.02, adjusted *P* value = 9.41 × 10^−4^), indicating the insertions for the genes are lower than expected according to the surrounding tetranucleotides. Figure 13 shows that the gene is similar to other genes labeled ‘Growth Defect’ or ‘Essential’ by the HMM+NP model. Rv3461c is a part of the L3P family of ribosomal proteins. Other genes in this family have been labeled as ‘Essential’ or ‘Growth Defect’ by the HMM+NP model and ‘Growth Defect’ per the Gene+TTN model. In fact, *rpmJ* was categorized as a ‘Growth Defect’ gene in early TraSH experiments (2). Therefore, this previously ‘Uncertain’ gene should be interpreted as ‘Growth Defect’, as the TTN-Fitness method suggests, with confidence.

These examples show the improvement of fitness assessment with the incorporation of tetranucleotides in an insertion-count-only model. This enables the TTN-Fitness method to account for the effect of genomic context on the Himar1 transposon insertion preferences and thus better assess a gene’s fitness defect due to genuine biological causes.

## DISCUSSION

Previous studies have demonstrated the presence of some site-specific biases on Himar1 transposon insertion preferences based on a nonpermissive pattern that exists around TA sites with low insertion counts in nonessential regions (8). This led us to hypothesize that perhaps insertion counts at different TA sites could be predicted based on surrounding nucleotides. We developed a model that captures nucleotide biases and uses them to predict changes in relative insertion counts, i.e., LFCs. The LFC metric compares raw counts at a site to the local average, which allows us to predict the deviation in insertion counts from the neighborhood rather than the absolute insertion counts themselves. This method allows us to examine just the effect of the nucleotides on the insertion counts, independent of biological effects (e.g., genes with different levels of growth defect). The STLM developed for the task incorporated tetranucleotides upstream and downstream of the TA site, taking advantage of the symmetric nature of the bias patterns observed in the heatmaps. Furthermore, the tetranucleotide features ensured that the model could capture nonlinear combinations (interactions) of nucleotides proximal to the TA site, not just incorporating the effects or individual nucleotides in an additive way. The STLM statistically performed as well as the neural network and in addition was able to provide further insight into nucleotide patterns that influence insertion counts.

The coefficients of the trained STLM showed that there was a pattern of insertion count suppression consistent with the nonpermissive pattern previously observed (8). In addition, a pattern of increased insertion counts in the presence of ‘A’ in the −3 position or ‘T’ in the +3 position was also visible. But the linear model represents these patterns in a more general way so that they can be used to predict expected insertion counts at any TA site, conditioned on the surrounding nucleotides. These nucleotide biases were able to explain up to ∼50% of insertion count variance in the other Himar1 data sets. These site-specific nucleotide biases were observed in a variety of TnSeq data sets from other mycobacterial and nonmycobacterial species. Comparing TA sites with substitutions in the ±4-bp window between two divergent strains of M. abscessus showed changes in observed LFCs that corresponded to nearby SNPs as predicted by the STLM, providing further evidence of the generality of these biases.

There is a precedent for transposons in some families having insertion biases for certain sequence patterns. Tc1 (also in the *mariner* family) was shown to weakly prefer inserting at TA sites with the consensus pattern CAYATATRTG (28). The pattern included a coupled symmetric target site preference of an ‘A’ in position −3 and a ‘T’ in position +3, consistent with our model. We were able to identify similar sequence-dependent patterns and quantify them in a more general way with a model that can predict expected insertion counts for every TA site. Another example of a transposon with an insertion bias is the Tn*5* transposase. It can insert anywhere in a genome but tends to insert in GC-rich regions (7, 29). A detailed pattern analysis applied to known Tn*5* insertion sites suggested that the consensus pattern for preferred target sites is AGNTYWRANCT (22). We were able to see this consensus pattern in our analysis of a Salmonella enterica Tn*5* TnSeq data set. Through this analysis, we were able to rule out the possibility that the biases we observe in Himar1 might be due to the generation of the TnSeq libraries rather than the transposon itself. We were also able to rule out possible effects of cellular factors, such as DNA-binding proteins, affecting transposon insertion locations, since all the libraries analyzed were generated via transfecting live cells rather than using *in vitro* (extracellular) transposition.

Early studies in E. coli suggested that the Himar1 transposon tends to insert at TA sites in more “bendable” regions of the genome (11), as measured experimentally. Bendability is a cumulative effect of specific nucleotides on local geometric parameters of the DNA helical axis; each nucleotide makes a small contribution, on the order of a few degrees, to angular distortion (bend, roll, tilt) of the axis, with different nucleotides (or combinations of nucleotides) having a different effect. This can accumulate over tens of nucleotides to produce a macroscopic bend or kink in the DNA. Goodsell and Dickerson (12) parameterized the geometric effects for each trinucleotide and used this to generate a model which can be used to predict the bend and twist of the helical axis accumulated locally using a sliding window. It was speculated that local bendability could facilitate the melting of the double helix, recognition/binding of the transposase, and formation of the precleavage complex (11). However, while it is possible that bendability contributes weakly to Himar1 insertion preferences, the effect likely spans a larger window of nucleotides than just ±4 bp around the TA sites; local bendability is not likely to be substantially affected by the 4 nucleotides on either side of the TA sites, which have a predominant influence according to our statistical analysis. In addition, we computed this around the TA sites in our data set and added it as a covariate in our linear models, but it did not improve the performance of the models.

**FIG 14:**
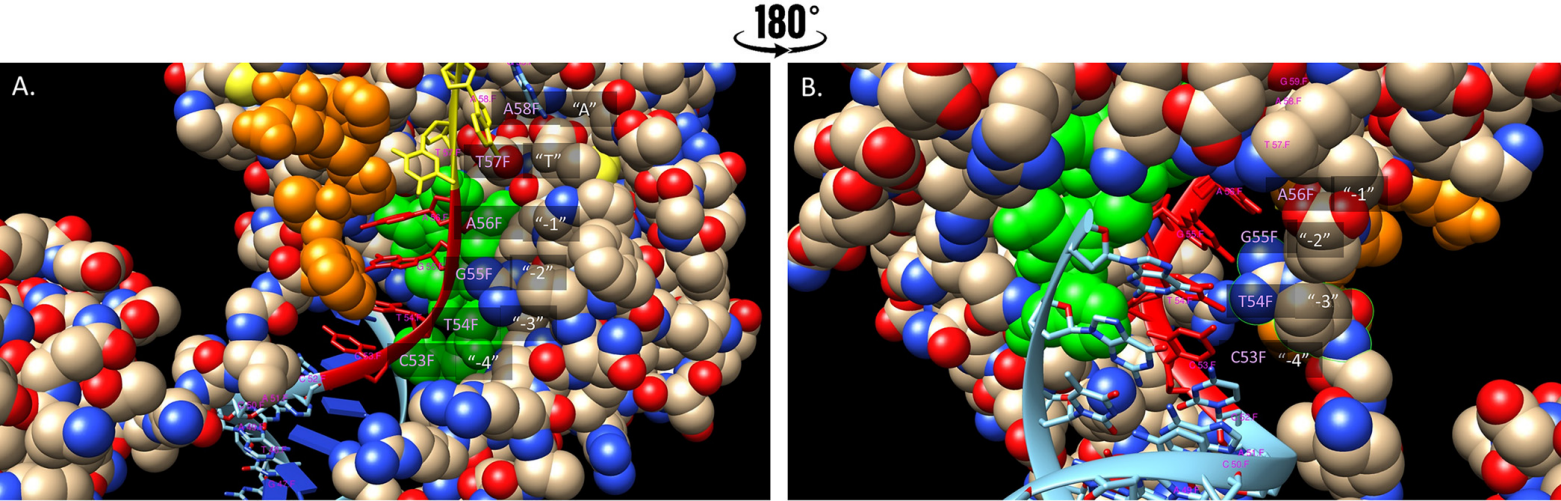
Crystal structure of the complex between the MosI transposon and DNA (PDB 4u7b). DNA double helix, with denatured (single-strand) end in the precleavage state. This is a stylized (cartoon) representation of the interaction. The red nucleotides represent C53-T54-G55-A56 (sites −4…−1). The yellow nucleotides represent the TA site, T57-A58. The blue nucleotide is G59, which is site +1. The transposase itself is shown as a molecular surface. Amino acids 119 to 124 (WVPHEL) are colored orange, and Asp284-His293 are colored green. The two images are vertical 180-degree rotations illustrating front and back views of the transposon-DNA interaction.

The patterns of nucleotide biases on Himar1 transposon insertion preferences may have emerged as a result of the physical interaction between the Himar1 transposase and the DNA. Figure 14 displays the X-ray crystal structure (PDB 4u7b) of the complex between the MosI transposon (also in the *mariner* family) and the precleavage state of the DNA double helix (30). As expected, the components of the TA dinucleotide (T57, A58) interact with the protein (residues 119 to 124 [WVPHEL], orange). However, the 4 adjacent nucleotides also make extensive contact with the protein in a small tunnel by packing against Asp284-His293 (green). Arg118 likely makes charged-polar interactions with the nucleotides at positions −2 and −3. These positions are where different nucleotides proximal to TA dinucleotides are observed to have insertion biases in Himar1 data sets. The interactions between these TA-adjacent nucleotides and amino acid side chains in the transposase could influence the energetics and therefore the frequency of successful transposon reactions at TA sites. While it would be tempting to try to perform a detailed analysis of the hydrogen bonding and other molecular interactions between nucleotides in the DNA fragment and amino acid side chains of the transposase they contact to derive a structural explanation for the observed preferences for certain nucleotides surrounding the TA site, it must be remembered that this structure is of MosI (whose insertion biases are unknown, except for TA restriction), and a detailed analysis of molecular interactions relevant to the biases of the Himar1 transposase, as we have characterized, will have to await determination of an X-ray crystal structure of a complex of the Himar1 transposase bound to a target DNA fragment (containing a TA site).

We demonstrated the utility of our model of nucleotide biases on Himar1 insertion frequencies by using it to improve gene essentiality predictions via the TTN-Fitness method. One way to determine the essentiality of a gene is to take the average count of insertions at all the TA sites in the gene and determine the essentiality based on a set of cutoffs (31). This method treats all TA sites as being equivalent *a priori* (i.e., as independent, and identically distributed observations, with equal prior probability of insertion) and does not allow for site-specific differences that can greatly affect the insertion count at each site. Incorporating these surrounding nucleotides takes out (or corrects for) the effect of insertion biases and focuses the analysis on true biological effects, thus increasing our certainty in fitness calls for these genes. In the TTN-Fitness method, we fit a linear model to the insertion counts at TA sites, incorporating the gene in which it resides and the nucleotides surrounding each site as covariates. The coefficients associated with genes in the regression model reflect how much the mean insertion counts in the gene deviate from the global average, after correcting for the expected insertion counts at each site in the gene. For most genes, predicted fitness did not change substantially between the ablative Gene-Only model and the Gene+TTN model of the TTN-Fitness method. However, the assessment for some notable genes did change with the inclusion of tetranucleotide features. For example, PE_PGRS13 was implied to be a ‘Growth Defect’ gene by a simplified insertion count based methodology (averaged at the gene level) due to the low insertion counts at its TA sites. However, sites in this gene are surrounded by mostly ‘G’s and ‘C’s which have been determined by Himar1 preferences to suppress insertions. So, the insertions are low, but they are expected to be low, and thus, the gene is determined to cause less of a fitness defect than previously predicted. The Gene+TTN model used in the TTN-Fitness method has an advantage for small genes with l≤3 TA sites (220 in H37Rv genome) such as Rv3461c (*rpmJ*), previously undetermined by essentiality estimates. The model is less susceptible to noisy counts (high or low) at individual sites because we can compare the observed counts at those sites to expected counts from their nucleotide context, correcting for the effect of insertion biases and thus improving the identification of conditionally essential genes and genetic interactions, i.e., to better distinguish true biological fitness effects by comparing the observed counts to expected counts using a site-specific model of insertion preferences. This method could also be helpful for analyzing differences in essentiality of genes between different strains (e.g., clinical isolates), where the TTN-Fitness model can correct for expected counts at TA sites to account for differences in the surrounding nucleotides (e.g., due to the different genetic backgrounds of the libraries).

## MATERIALS AND METHODS

### Data set of 14 independent Himar1 insertion libraries of M. tuberculosis H37Rv grown *in vitro*.

We obtained 14 independent TnSeq libraries in M. tuberculosis H37Rv previously analyzed (8), representing a combined total of 35,314,576 independent insertion events by the Himar1 transposon. All libraries were treated uniformly and grown in standard laboratory medium (7H9/7H10). Briefly, the 14 libraries were constructed by transfection of the MycoMar T7 phagemid carrying the Himar1 transposon into the parent strains. DNA was extracted for sequencing and fragmented by shearing. Adapters were then ligated, and the transposon-genomic junctions were PCR amplified (4). The M. abscessus, M. avium, M. smegmatis, *Caulobacter*, Rhizobium leguminosarum, and M. tuberculosis H37Rv ΔRv0060 libraries were constructed similarly. Every library in the 14 replicates has a mean saturation, i.e., percentage of TA sites in the genome with 1 or more transposon insertions, of 0.65, totaling to a saturation of 0.85 for the entire data set. As these are 14 independent libraries, the probability of a nonessential site with zero insertions for stochastic reasons is quite small. However, there is a lack of insertions in nonpermissive sites in nonessential regions, which account for 9% of all TA sites. Most of the remaining sites with zero insertions correspond to essential regions.

This high level of saturation enabled us to reliably observe the nucleotide bias of insertion counts at different TA sites. The data set was normalized using TTR normalization in TRANSIT (9) (the top and bottom 5% of read counts are trimmed to reduce the influence of outliers). Counts are then divided by the total counts in each data set and scaled back up so the mean count at nonzero sites is 100.0. We identified essential regions as consecutive sequences of 6 or more TA sites with counts of two or less and subsequently removed them. Using the resulting data set, we were able to explain nucleotide bias at TA sites not only for H37Rv but also for other mycobacterial and nonmycobacterial Himar1 TnSeq data sets.

### Significance of the correlation of insertion counts between TnSeq data sets.

The correlation of insertion counts at TA sites between TnSeq data sets was calculated using a Pearson correlation coefficient. As mentioned previously, the log of insertion counts was used, since the Pearson correlation coefficient assumes that the input data are normally distributed. The two-tailed *t* test for the means for two independent samples was used to measure whether the expected value differs significantly between samples. Since we do not assume population variance between the two data sets is equal, the Welch *t* test is performed.

### Tenfold cross-validation linear regression.

The data were split for 10-fold cross-validation using sklearn.model_selection.KFold(). Within these folds, we used Ridge Regression implemented in sklearn.Ridge() with alpha = 0.1 to train and test linear models (target values of log insertion counts or LFCs) with L2 regularization.

### Hyperparameter tuning the neural network.

The data were separated into training and testing using a 70-30 train-test split. We used 10-fold cross-validation on the 70% training split of the data to tune the number of nodes per hidden layer, number of hidden layers, the activation function, value of alpha, and whether to use early stopping. We used scikit-learn’s GridSearchCV to perform this operation and checked the accuracy of the final hyperparameters set on the reserved 30% set.

### Mean LFCs per nucleotide-position pair.

For every position ±20 bp from the TA site, we filtered for nucleotides ‘A’, ‘T’, ‘C’, and ‘G’. We took the mean LFC of the training samples (TA sites) with that nucleotide in that position. This calculation yielded the mean LFC for each nucleotide at each position 20 bp from the TA site, which was then visualized as a heatmap with a diverging color palette.

### Model adjustment calculations.

Each TnSeq data set has a slightly different LFC distribution. Thus, the predictions of a TnSeq data set from the STLM, trained on H37Rv data, had to be adjusted. This was accomplished by a simple regression-based procedure. First, we determined the linear relationship between the mean LFC for each tetranucleotide in H37Rv by regressing it against the mean LFC of each tetranucleotide in our target strain. The linear relationship could be represented as *targetStrainLFCs* = *m* × *H37RvLFCs* + *offset*. We used this relationship to adjust the LFC predictions made by the STLM using the target strain’s data *LFC_adjusted_* = *m* × *LFC_STLM_* + *offset*.

### Average change in observed LFC versus average change in predicted LFC between strains.

In comparing the genome sequences of M. abscessus ATCC 19977 and the Taiwan49 clinical isolate, there are 9,303 TA sites with exactly one SNP in the surrounding ±4-bp window. There are 8 positions and 12 possible substitutions per position, thus 96 possible SNPs that can occur. For each of these possible nucleotide changes, we calculated the difference between the observed LFC in the reference strain and the observed LFC in the isolate strain. The mean of this difference was determined to be the mean observed LFC difference for that SNP. We performed a similar calculation for the predicted LFCs. Using the STLM, we found the predicted LFCs at TA sites in the reference strain and predicted LFCs at TA sites with a specific SNP in the clinical isolate. The average of the difference in these two predicted LFCs was the mean change in predicted LFC.

### Using the binomial distribution to filter small essential genes before training the Gene+TTN model.

The first step in fitness estimation is to identify and remove any essential genes. These genes are excluded from the Gene+TTN analysis. First, larger essential genes (with ≳10 TA sites) are identified using the Gumbel method in TRANSIT (9). Then, smaller essential genes with no insertions are identified and removed based on a binomial calculation. Given the probability that an insertion does not occur (*P* = 1.0- saturation), the probability of *k* TA sites out of *n* total having no insertions follows the binomial distribution:

$$P\left(k\right)=\left(\begin{array}{c} n\\ k \end{array}\right)p^{k}q^{n-k}$$

Thus, the probability that all TA sites in a gene have 0 insertions is a binomial distribution where *k* = *n*:

$$P\left(n\right)=\left(\begin{array}{c} n\\ n \end{array}\right)p^{n}{\left(1\;-\;p\right)}^{0}\;=\;p^{n}$$

We use this formula to determine the minimum *n* such that *P*(*n*) is <0.05, and we then label any genes with *n* or more TA sites, all of which have insertion counts of 0, as ‘Essential-B’ (‘ESB’). This method is a necessary additional step to the Gumbel method to find smaller genes that may have been missed, especially in data sets with lower saturation.

### Data availability.

The source code (Python scripts) for performing the calculations described in this paper (including the TTN-Fitness model) is available at https://github.com/ioerger/TTN-Fitness. The raw data files (wig files with insertion counts at TA sites) for the 14 replicate libraries of M. tuberculosis, along with 3 replicates for M. abscessus Taiwan49, can also be found in the demodata/directory of the same GitHub repository.
