# Supplementary figures and images for: Modeling Site-Specific Nucleotide Biases Affecting Himar1 Transposon Insertion Frequencies in TnSeq Data Sets
Source: mSystems. 2021 Oct 19;6(5):e00876-21. doi: 10.1128/mSystems.00876-21 (PMC8525568; doi:10.1128/mSystems.00876-21)

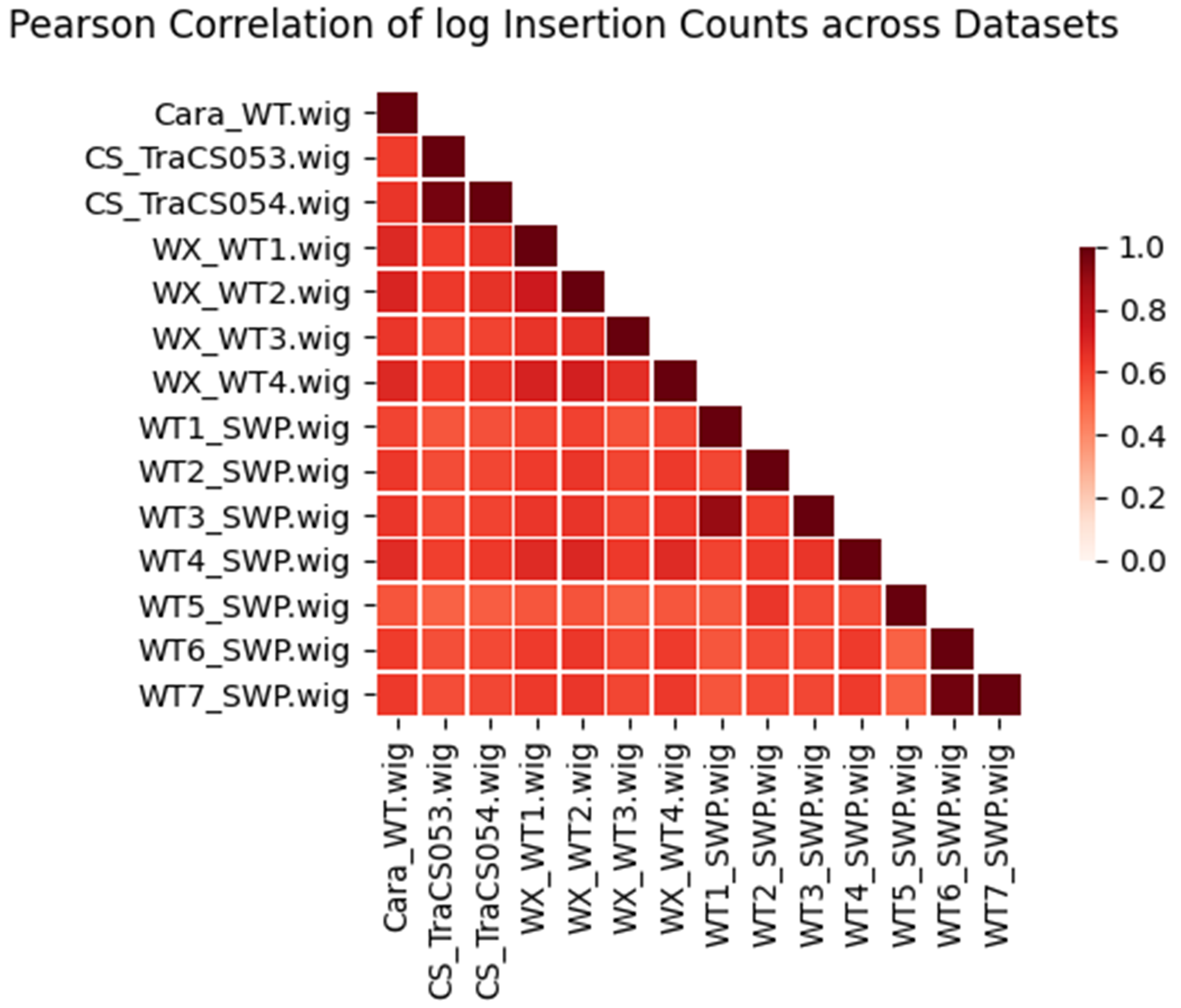

Supplement: FIG S1 [file msystems.00876-21-sf001.tif]

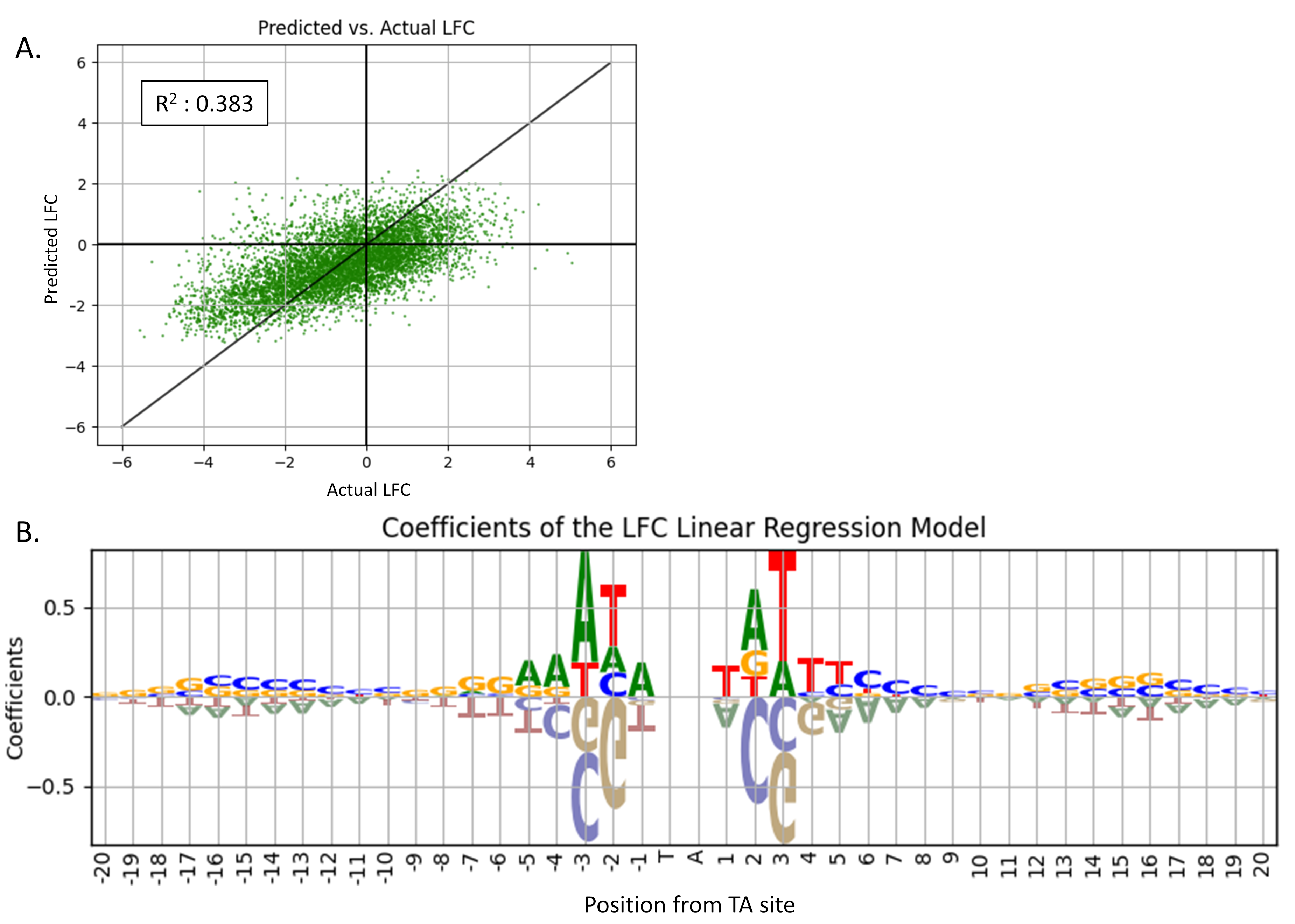

Supplement: FIG S2 [file msystems.00876-21-sf002.tif]

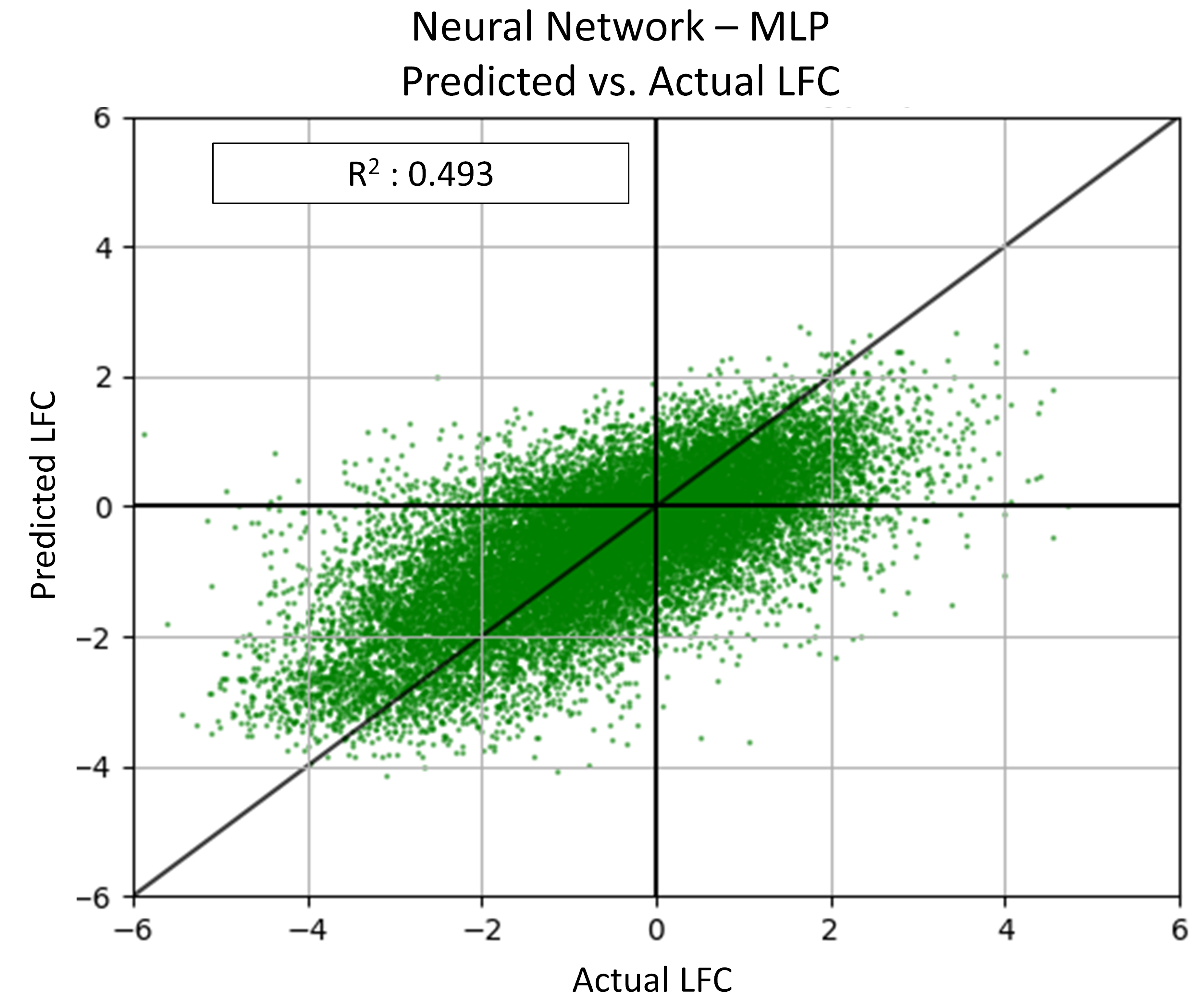

Supplement: FIG S3 [file msystems.00876-21-sf003.tif]

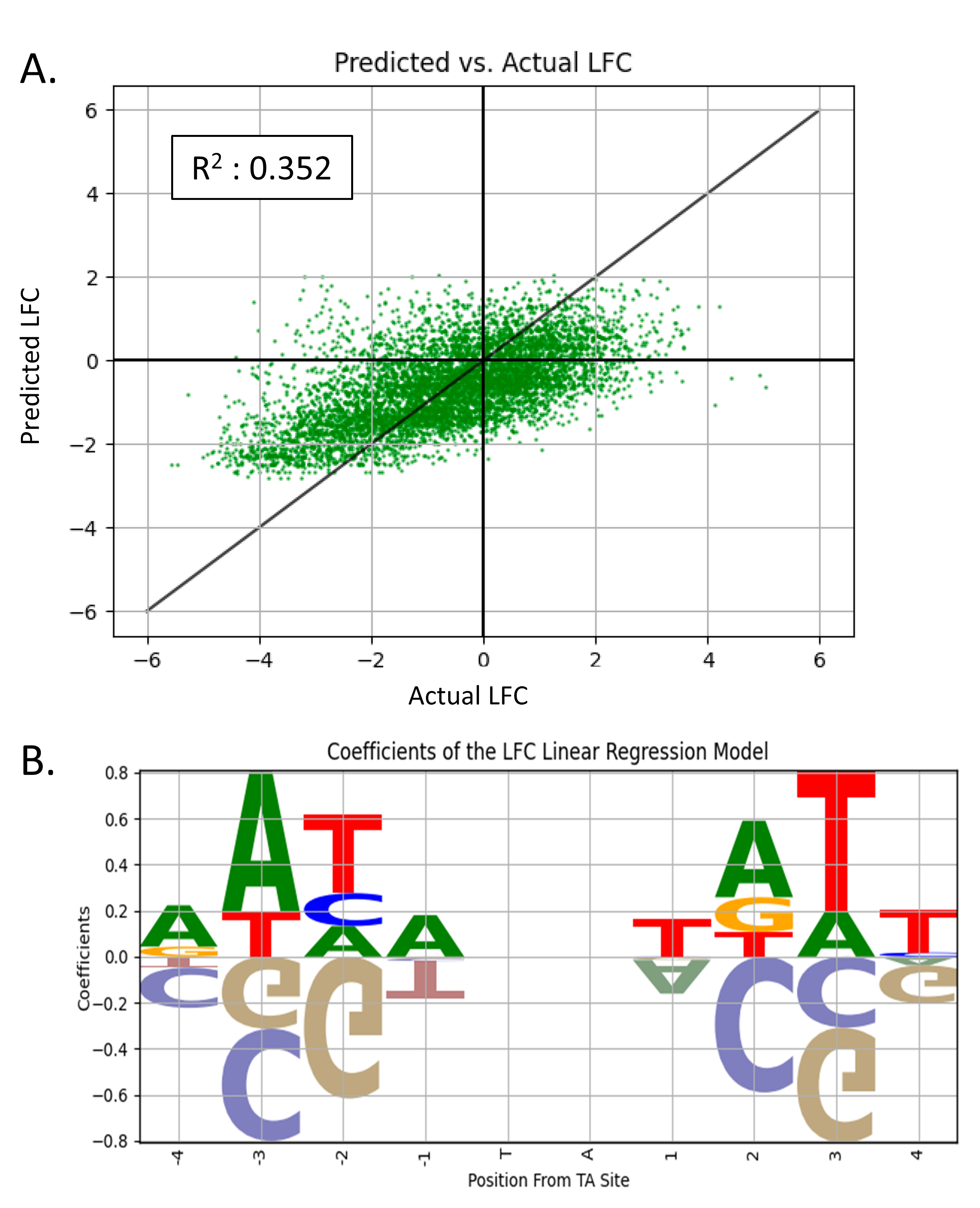

Supplement: FIG S4 [file msystems.00876-21-sf004.tif]

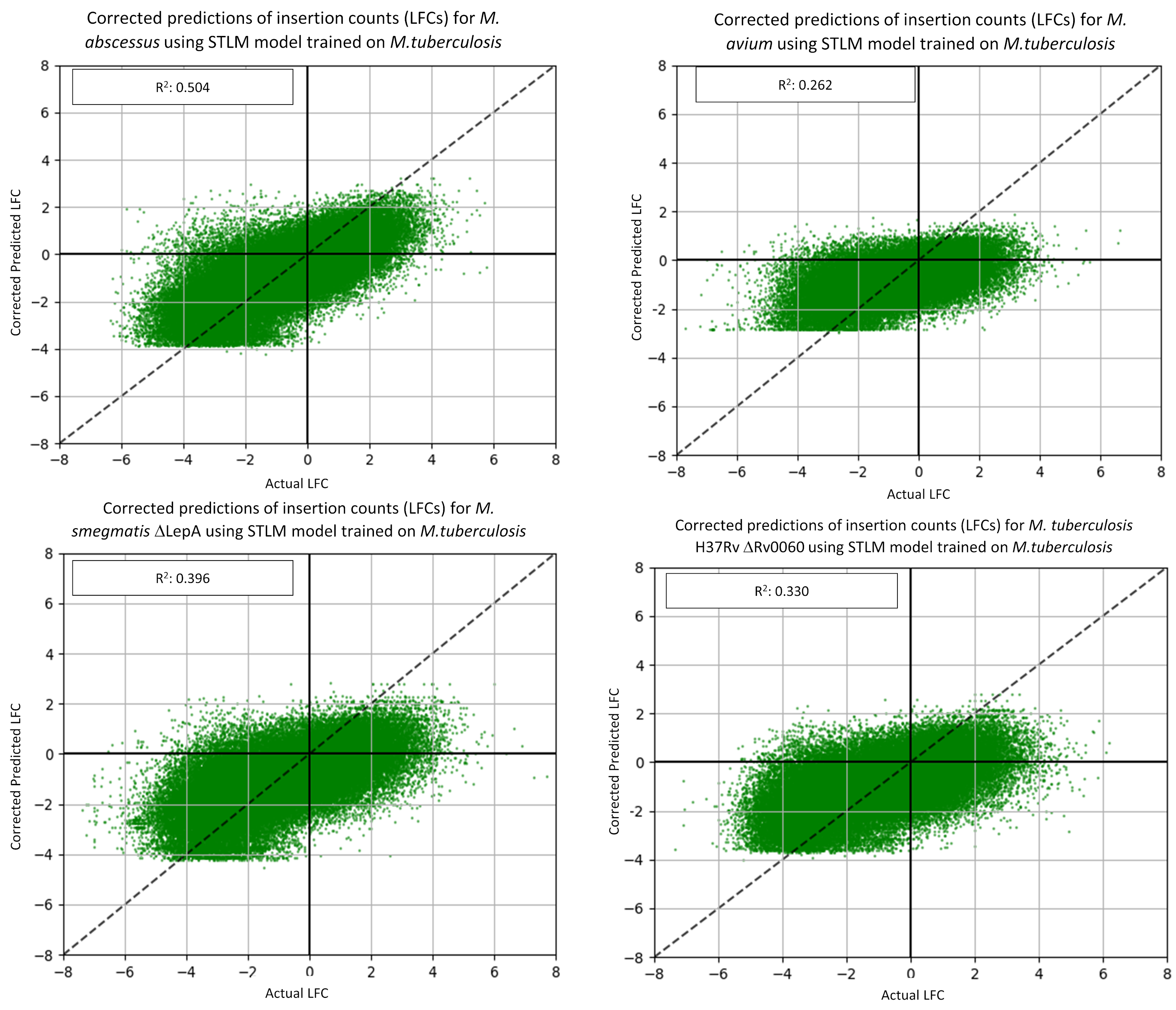

Supplement: FIG S5 [file msystems.00876-21-sf005.tif]

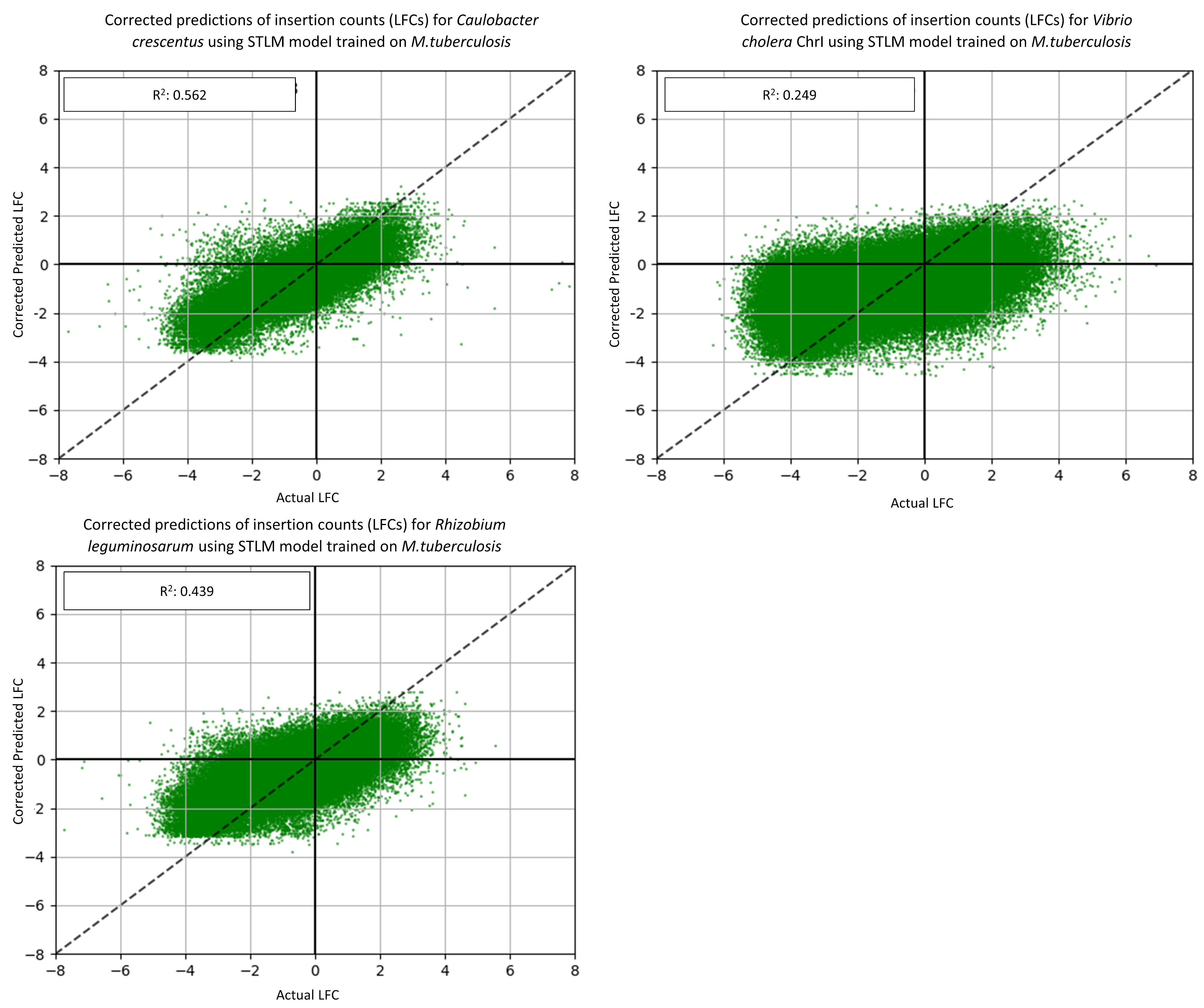

Supplement: FIG S6 [file msystems.00876-21-sf006.tif]

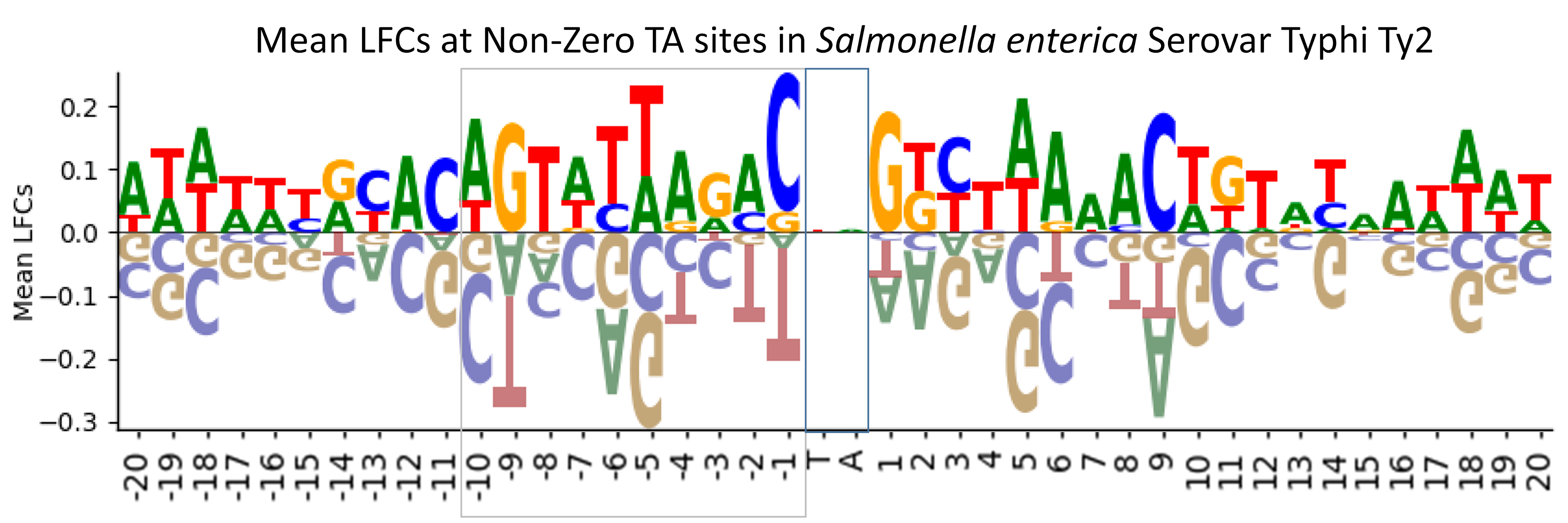

Supplement: FIG S7 [file msystems.00876-21-sf007.tif]
